# The Emotional Gatekeeper: A Computational Model of Attentional Selection and Suppression through the Pathway from the Amygdala to the Inhibitory Thalamic Reticular Nucleus

**DOI:** 10.1371/journal.pcbi.1004722

**Published:** 2016-02-01

**Authors:** Yohan J. John, Basilis Zikopoulos, Daniel Bullock, Helen Barbas

**Affiliations:** 1 Neural Systems Laboratory, Department of Health Sciences, Boston University, Boston, Massachusetts, United States of America; 2 Human Systems Neuroscience Laboratory, Department of Health Sciences, Boston University, Boston, Massachusetts, United States of America; 3 Graduate Program for Neuroscience, Boston University and School of Medicine, Boston, Massachusetts, United States of America; 4 Department of Psychological and Brain Sciences, and the Center for Computational Neuroscience and Neural Technology, Boston University, Boston, Massachusetts, United States of America; Radboud Universiteit Nijmegen, NETHERLANDS

## Abstract

In a complex environment that contains both opportunities and threats, it is important for an organism to flexibly direct attention based on current events and prior plans. The amygdala, the hub of the brain's emotional system, is involved in forming and signaling affective associations between stimuli and their consequences. The inhibitory thalamic reticular nucleus (TRN) is a hub of the attentional system that gates thalamo-cortical signaling. In the primate brain, a recently discovered pathway from the amygdala sends robust projections to TRN. Here we used computational modeling to demonstrate how the amygdala-TRN pathway, embedded in a wider neural circuit, can mediate selective attention guided by emotions. Our Emotional Gatekeeper model demonstrates how this circuit enables focused top-down, and flexible bottom-up, allocation of attention. The model suggests that the amygdala-TRN projection can serve as a unique mechanism for emotion-guided selection of signals sent to cortex for further processing. This inhibitory selection mechanism can mediate a powerful affective ‘framing’ effect that may lead to biased decision-making in highly charged emotional situations. The model also supports the idea that the amygdala can serve as a relevance detection system. Further, the model demonstrates how abnormal top-down drive and dysregulated local inhibition in the amygdala and in the cortex can contribute to the attentional symptoms that accompany several neuropsychiatric disorders.

## Author Summary

Emotional experiences grab our attention. Information about the emotional significance of events helps individuals weigh opportunities and dangers to guide goal-directed behavior, but may also lead to irrational decisions when the stakes are perceived to be high. Which neural circuits underlie these contrasting outcomes? A recently discovered pathway links the amygdala—a key center of the emotional system—with the inhibitory thalamic reticular nucleus (TRN) that filters information between the thalamus and cortex. We developed a neural network model—the Emotional Gatekeeper—that demonstrates how the newly discovered pathway from the amygdala to TRN highlights relevant information to help assess threats and opportunities. The model also shows how the amygdala-TRN pathway can lead normal individuals to discount neutral but useful information in highly charged emotional situations, and predicts that disruption of specific nodes in this circuit underlies distinct psychiatric disorders.

## Introduction

To survive in a dynamic and information-rich environment, individuals must flexibly allocate attention to emotionally salient stimuli. Individuals must be sensitive to environmental opportunities as well as threats. Bottom-up attention to the most salient stimulus is often complemented by top-down attention to stimuli that are relevant to one’s goals. When there is no strong motivation or bias, the current or most recent salient stimulus may guide behavior. But sometimes it is important to suppress bottom-up salience signals. For example, if an animal is extremely hungry, it may be necessary to shift focus away from stimuli that are predictive of predators and restrict focus to predictors of food. Conversely, caution is often more important and attention must be restricted to predictors of danger. Neural circuitry must therefore facilitate adaptive links between emotional information, behavioral plans, and attention.

Affective information has well-known effects on attention and decision-making [1]. Stimuli that are associated with strongly positive or strongly negative experiences attract attention and bias the cognitive faculties of humans and animals [2,3]. Such stimuli can also render organisms less sensitive to other aspects of the environment, preemptively locking out other signals and potentially leading to “emotion-induced blindness” [4]. These widely observed behavioral tendencies suggest that the effect of emotions on attention and other cognitive processes is not purely an amplification of salient signals, but also involves strong suppressive mechanisms.

The question arises of how emotion-related neural signals can mediate both the selective and suppressive aspects of attention. A potentially powerful pathway for emotion-guided attention was recently discovered in the primate brain: a robust projection from the amygdala to the thalamic reticular nucleus (TRN) [5]. This pathway was previously unknown in any species. The amygdala is a major hub of the emotional system, and is central for assessing the affective salience of stimuli and contexts and transmitting this information to other regions [6]. Just as the amygdala is a hub for emotional processing, the inhibitory TRN is a critical hub for attentional processing [7,8]. Crick [9] suggested that the distinctive position of TRN as the primary inhibitor of the sensory thalamus makes it the proximal controller of the “attentional searchlight”. Physiological and behavioral studies support such a role for the TRN. Loss of TRN neurons in humans and rodents results in attentional deficits [10,11]. The TRN appears to provide an early common path for gating the flow of signals between thalamus and cortex. The strong excitatory pathway from the amygdala to TRN suggests that the brain’s emotional hub can exert direct control over a strong inhibitory hub of the attentional system. This direct linkage may serve as an emotional ‘gatekeeper’ that uses affective salience to direct and shift attention, regulating the passage of information from thalamus to cortex.

The goal of this computational modeling study was to investigate the possible mechanisms through which the amygdala-TRN pathway influences attention. We show how the circuit linking amygdala, TRN, thalamus and cortex enables allocation of both flexible bottom-up and focused top-down attention guided by emotions. Bottom-up attention allows an animal to focus on the most salient aspects of the environment and choose a plan accordingly. Top-down attention allows an animal to focus only on what is relevant to a pre-selected plan. In our model, the Emotional Gatekeeper (EmGate), the local amygdala circuit forms associations between sensory stimuli and affective consequences, resulting in affective salience signals. These salience signals influence TRN-dependent attentional selection and suppression, and also provide ‘evidence’ for the system to decide on an action. The model indicates that the amygdala-TRN pathway can mediate selection of emotionally salient stimuli and block or weaken transmission of non-salient or irrelevant signals to cortex, thereby constraining the information available for subsequent cortical processing. The model also predicts that inhibitory interneurons in the amygdala and in prefrontal cortex perform crucial and complementary resetting functions that allow for flexible shifting of attention and flexible decision-making. By extension, the excitatory inputs to these interneurons regulate flexibility. We hypothesize that expectation-related signals—both expectation confirmation and expectation violation—are necessary to ensure that emotion-based attention and decision-making can shift in accord with the current situation. The contribution of inhibitory interneurons to flexibility is a testable prediction of the model. The model also predicts that three mechanisms can contribute to abnormal emotional-related behavior: dysfunctional cortical drive to the amygdala, dysregulation of local inhibition in the amygdala, and dysregulation of local inhibition in the cortex. These mechanisms likely contribute to the attentional symptoms of some neuropsychiatric disorders and to the transient warping of decision-making by normal individuals when confronted with high-stakes emotional situations. From a computational perspective the EmGate model also illustrates how three different sources of inhibition—in the TRN, cortex, and amygdala—facilitate flexible competition between corticothalamic loops.

## Model

### Background for model

The model rests on three key findings about the interactions between amygdala, dorsal thalamus, cortex, and the inhibitory TRN: (i) The amygdala mediates affective learning; (ii) The amygdala sends strong excitatory projections to TRN; and (iii) The TRN sends off-surround inhibitory projections to the thalamus, facilitating competition between cortico-thalamic loops, as elaborated below.

#### (i) The amygdala mediates affective learning

The amygdala has been viewed as central to positive (appetitive) and negative (aversive) learning and response [12,13], as well as having a key role in motivation, memory and cognitive-emotional interactions [14,15]. The pertinent parts of the amygdala for the model include the basolateral group (BLA), consisting of the basal amygdala (BA) and the lateral (LA) nucleus. The BA has its strongest bidirectional connections with limbic prefrontal cortices (PFC) that include the posterior orbitofrontal cortex (pOFC) and the anterior cingulate cortex (ACC) in primates and the analogous prelimbic and infralimbic cortices in rodents [16,17]. Synaptic changes in LA mediate learned associations between an unconditioned stimulus (US) and a conditioned stimulus (CS). Pavlovian fear conditioning studies in rodents suggest involvement of BLA as a whole in appetitive learning [18–23], based on neuronal responses to the positive and negative value of stimuli [24]. In the primate amygdala neuronal responses also correlate with spatial attention to positive and negative stimuli [25].

#### (ii) The amygdala sends strong excitatory projections to TRN

The TRN can be divided into topographic sectors that receive projections from cortical areas and have bidirectional connections with the corresponding dorsal thalamic nuclei. Thus, there are sensory and motor TRN sectors and a PFC sector that is connected with the mediodorsal thalamic nucleus (MD). Unlike the projections from sensory cortices to TRN, some parts of lateral and orbital PFC and MD do not restrict their projections to their dedicated (frontal) sector, but also innervate the somatosensory, visual and auditory sectors of TRN [26,27]. The amygdala is connected with both MD and pOFC [16,28–30], and appears to contribute to cognitive-emotional interactions [14,16,31]. This tripartite circuit raised the possibility that the amygdala also projects to TRN, a hypothesis that was confirmed [5]. Pathways mostly from BA form large synapses with TRN neurons. About 70% of the axon boutons from the amygdala terminate in the rostral sector of TRN, which also receives prefrontal cortical projections. Thus, a significant proportion, about 30%, of amygdalar projections terminate more caudally, within the sensory sectors of TRN. Of the TRN projections from pOFC, MD and amygdala, the amygdalar pathway has the highest proportion of large boutons (20%). Amygdalar axon boutons are also the most proximal to the soma of TRN neurons.

#### (iii) The TRN sends off-surround inhibitory projections to thalamus, facilitating competition between cortico-thalamic loops

**Fig 1:**
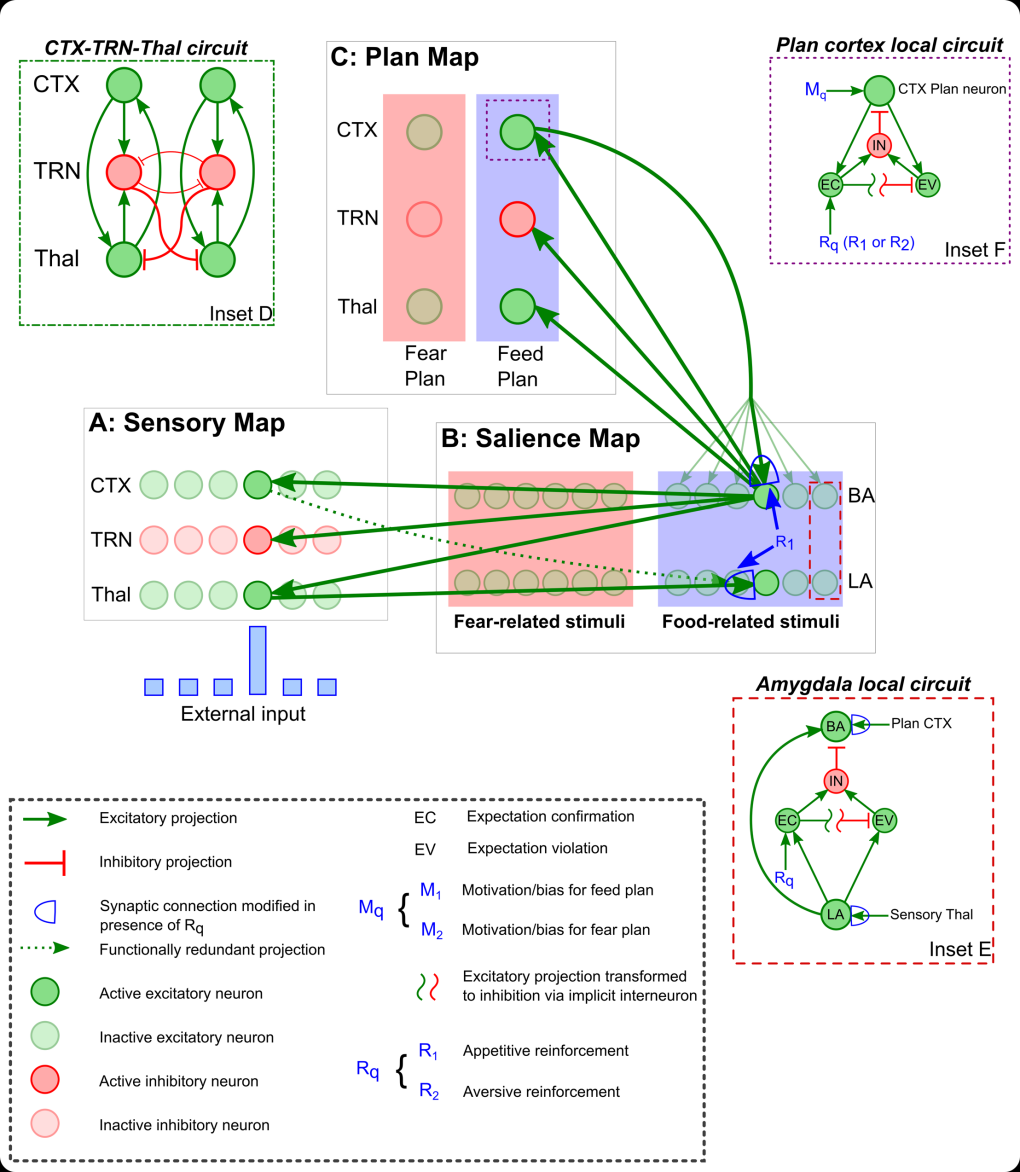
Schematic diagram of the EmGate model. ***A–C*,** The model consists of three interacting maps: a sensory map (A), a salience map (B), and a plan map (C). The sensory map (A) and the plan map (C) are instantiations of the basic cortex-TRN-thalamus circuit shown in Inset D. This circuit enables competition between parallel cortico-thalamic loops (green excitatory projections). Each loop can inhibit all others through off-surround inhibition mediated by the TRN (red inhibitory projections). In the sensory map (A), this competition is equivalent to selective attention. In the plan map (C) this competition is equivalent to decision-making. The salience map (B) is the amygdala circuit, which represents sensory stimuli that acquire affective salience. Two subpopulations of LA and BA neurons (green excitatory neurons) exist in the salience map (B): one devoted to labeling appetitive stimuli (blue rectangle), and one devoted to labeling aversive stimuli (pink rectangle). Synaptic connection weights linking sensory thalamus with the feed-related LA subpopulation are enhanced when sensory thalamic activity co-occurs with food-related reinforcement signal R_1_ (in B and Inset E). Similarly, synaptic connection weights linking sensory thalamus with the fear-related LA subpopulation are enhanced when sensory thalamic activity co-occurs with fear-related signal R_2_. LA conveys salient sensory signals to BA, which communicates bidirectionally with the plan map (C). Each BA subpopulation sends converging signals to the corresponding plan loop, providing ‘evidence’ in support of the corresponding plan. Each BA subpopulation also receives top-down projections from the corresponding plan cortex neuron. Connections between plan cortex and BA are also strengthened by the reinforcement signals R_q_ (R_1_ or R_2_; in B and Inset E). The local amygdala circuit is shown in Inset E. Each BA neuron receives inhibition from a local interneuron that is excited by two signals: fast expectation confirmation (EC) and slower expectation violation (EV). The EC signal also inhibits EV preventing it from building up. The EC and EV signals are dependent on LA activity and reinforcement signals. The local plan cortical circuit is shown in Inset F. Each plan cortical neuron also receives inhibition from a local interneuron. The EC and EV signals in cortex are computed locally based on plan cortex activity and reinforcement signals. Each plan cortical neuron also receives a top-down motivation or biasing signal, M_q_ (M_1_ or M_2_ in Inset F). The two sources of inhibition in the Plan and Salience Map local circuits that are driven by local EC and EV signals can interrupt persistent positive excitatory feedback, allowing the system to reset itself and remain sensitive to changing contingencies. The expectation represented by each LA neuron is stimulus-specific and the expectation represented by each plan cortical neuron is plan-specific. Stimulus specificity is lost at the level of the Plan Map because all BA signals within a subpopulation converge onto a single plan loop, providing a summed excitation to the corresponding plan cortex, plan TRN and plan thalamus neurons. The BA neurons project to the sensory map (A), cortex (CTX), TRN and thalamus (Thal), biasing the competition among sensory loops in favor of stimuli that have been labeled by LA as salient. This allows the amygdala to bias attention to sensory signals with affective salience.

The amygdala-TRN projection provides a robust mechanism for affective information to influence the dynamics of the circuit linking TRN, thalamus and cortex. The entirely inhibitory TRN receives excitatory glutamatergic projections from thalamus and cortex and sends inhibitory projections to the dorsal thalamus (Fig 1, Inset D). Neurons in TRN also innervate each other, facilitating lateral inhibition. Off-surround lateral inhibition between TRN neurons enhances contrast in the competition between cortex-TRN-thalamus (CRT) loops, allowing small differences in input to the TRN to contribute more effectively to the competition process. The connectivity of TRN suggests that it can serve as an inhibitory gate that selectively regulates the flow of signals from thalamus to cortex [32,33]. The regions that send projections to TRN influence how and when the gate is opened or closed, and thus may serve as gatekeepers. The amygdala appears to be one of the most powerful gatekeepers, given the strength and proximity of innervation of TRN neurons. The topographic nature of projections to TRN and between cortex and thalamus suggests that the cortex-TRN-thalamus (CRT) circuit can be thought of as a band of parallel loops or ‘channels’ (Fig 1).

The possible ways for the TRN to innervate the thalamus occupy a spectrum from ‘closed-loop’ patterns, in which each TRN neuron inhibits the same thalamic neurons that excite it, and ‘open-loop’ patterns, in which each TRN neuron projects in an off-surround manner, fanning out to inhibit thalamic neurons that do not reciprocate with excitation. Much of the evidence suggests that open-loop connectivity is predominant [27,34,35].

Open-loop off-surround inhibition of thalamus by TRN is ideally suited to mediate selective attention via competition between cortico-thalamic loops [35–37]. Levels of acetylcholine and norepinephrine during active wakefulness tend to prevent the CRT circuit from entering oscillatory modes seen during sleep [38–41]. Our focus here is attentional processing during active awake behavior, so we employ the tonic mode and open-loop TRN-thalamic connectivity in the EmGate model.

### Model design

The EmGate model is a neural network model that illustrates how emotion-related amygdalar processes can interact with TRN-modulated attention in both top-down and bottom-up ways. The model integrates the anatomical and physiological findings reviewed above. We assume that the circuit formed by cortex, thalamus and TRN serves as an on-center off-surround network [42,43] and gates the transmission of signals from thalamus to cortex. The inhibitory projections from TRN to thalamus and between TRN neurons serve as the off-surround component of the network, while the mutual excitatory connections between the thalamus and cortex serve as the on-center component. Inhibition from TRN mediates a competition that determines which cortico-thalamic loops are allowed to reverberate, and which are silenced. For loops that respond to sensory input, competition leads to selective attention. For loops that control or modulate action and planning, competition mediates decision-making. We employed instances of the CRT circuit for both of these functions. The basic CRT connectivity is represented schematically in Fig 1 (Inset D).

The EmGate model, represented schematically in Fig 1, consists of three interacting subsystems: (1) a sensory map that represents stimulus information; (2) a salience map that assigns affective value (positive or negative) to signals received from the sensory map; and (3) a plan map that allows signals received from the salience map to serve as ‘evidence’ that drives particular behavioral responses. The sensory map and the plan map are each instantiations of the basic CRT circuit, and the salience map is based on the local circuit in the amygdala.

#### Sensory map

The sensory map may be located either in unimodal sensory CRT loops or in high order loops such as those created by PFC, MD and the rostral TRN sector. There is considerable functional heterogeneity in PFC subregions, with groups of neurons responding to sensory inputs, goals, affective states, motor outputs, and combinations thereof. Some neurons in dorsolateral PFC, for example, respond primarily to sensory signals [44,45]. Since the amygdalar BA projects to both the rostral TRN as well as to sensory sectors of TRN, it is in a position to filter sensory signals in both unimodal and high order CRT loops [5]. The sensory map can be understood as a one-dimensional feature map, such as an auditory frequency map arranged tonotopically. For each feature there is a corresponding model neuron in sensory thalamus, TRN, and cortex. Each model neuron represents a group of neurons with similar response properties and connectivity. Notably, there are more neurons per ‘channel’ in cortex, but the neurons in a particular cortical column have similar receptive fields and connectivity.

#### Salience map

Sensory signals arrive at LA and are relayed to BA, which is reciprocally connected with pOFC [16,31,46,47]. The sensory cortical and sensory thalamic neurons are strongly correlated as a result of the paradigms simulated here, and therefore we omit projections from sensory cortex to LA, which provide information that is functionally redundant for current purposes. These projections are shown as a dotted line (Fig 1A and 1B). LA projects to pOFC, but in primates it receives very weak projections from pOFC compared with the BA [48,49]. The model assumes that the weak projections from pOFC to LA do not contribute significantly to LA activity compared with sensory signals from thalamus. This simplification facilitates a functional separation between LA and BA that contributes to the model’s mechanism for attentional flexibility, as discussed further below.

In addition to neutral sensory signals, the amygdala also receives signals that represent USs, such as an airpuff or food reward [50,51]. In the EmGate model, the amygdalar LA and BA nuclei are each divided into two subgroups of neurons: one for appetitive CS-US associations, and one for aversive CS-US associations [24]. In Fig 1B these two subgroups are depicted as segregated for clarity, but they may also be intermingled. The stimuli are mapped topographically from sensory thalamus to each LA subgroup [19,52], which in turn are mapped topographically to the corresponding BA subgroup. The BA principal neurons serve as the output neurons of the amygdala to cortex. Topographic projections from each BA subgroup are sent to the thalamus and TRN of the sensory map.

Bidirectional connections between each BA subgroup (appetitive and aversive) and its corresponding cortical plan—exemplified here by ‘feed’ and ‘fear’ plans—are all-to-all. Through learning, any CS that is paired with a US will excite the corresponding plan in a bottom-up manner via the projection from BA to the plan thalamus. For example, repeated pairing of a CS with food reward will strengthen the synaptic weights that link sensory thalamus to appetitive LA, and those that link the feed plan cortex to BA. Because of synaptic strengthening, the next time the CS is presented, the projections from BA will trigger the feed plan. Bottom-up processing allows the model to respond to incoming stimuli based on their current affective salience. The projection from plan cortex to BA allows for recurrent excitation. If the aversive plan CRT is preferentially excited through top-down modulation from upstream areas, then the plan cortex will excite BA representations of the stimulus that was previously paired with an aversive US. This boost in top-down excitation will in turn cause selective enhancement of the sensory map representations of the stimulus that was previously paired with an aversive US, through BA projections to the sensory CRT. Off-surround inhibition suppresses thalamic responses to stimuli that are not consistent with the pre-selected top-down plan, preventing these stimuli from having appreciable effects on sensory cortex.

In addition to inter-areal projections, the EmGate model employs local inhibitory interneurons that form synapses with BA principal neurons [53]. Each BA principal neuron is inhibited by a corresponding interneuron. The interneurons allow BA to automatically reset itself and remain sensitive to changing reinforcement contexts. Two types of resetting are incorporated into the model: a fast reset after a successful prediction, and a slower reset after unsuccessful prediction. In each subpopulation, the activity of each LA neuron serves as the expectation signal for the corresponding stimulus. Thus activity of each LA neuron represents a stimulus-specific expectation, or a prediction that a stimulus will be followed by a US. If the expected US arrives while an LA principal neuron is active, the corresponding interneuron becomes excited, inhibiting the corresponding BA principal neuron.

Expectation confirmation signals cannot be the sole source of excitation to the inhibitory interneurons. A second source of excitation to the interneurons is needed, which will prevent attentional fixation in the event of unexpected omission of reinforcement. We therefore posit a second source of excitation to the interneurons that is triggered by unsuccessful prediction, or expectation violation. Once an LA neuron becomes active, an expectation violation signal begins to build up. This expectation violation signal excites the corresponding interneuron, triggering inhibition of the corresponding BA neuron. The build-up of the expectation violation signal is counteracted by inhibition from the expectation confirmation signal, preventing a redundant burst of inhibition from the interneuron. Thus, both confirmation and violation of expectation are important inhibitors of BA activity.

Attentional flexibility requires that the expectation signal that triggers confirmation or violation be stimulus-specific and triggered by the actual presence of the stimulus in the environment. For this reason we assume that LA activity does not reflect top-down influence from plan cortex. With this assumption in place LA activity can provide an expectation signal that is not ‘contaminated’ by top-down expectation. Because BA participates in a positive feedback loop with the plan map, it is activated not only by input from LA, but also by plan-related activity from cortex. This means that BA activation is not as reliable an indicator of the presence of a salient stimulus as LA activity, which we assume is solely activated by sensory thalamic input. Unlike LA, BA activity reflects not only the presence of affectively labeled sensory signals, but also top-down modulation from plan cortex. This additional source of input to BA allows the BA-TRN projection to direct sensory attention even if the salient stimulus is not currently being presented. The above assumptions are consistent with the predominant pathways from prefrontal cortices to the amygdala [49]. However, from a computational perspective the key functional separation required for the behavior of the model does not have to be between BA and LA, but between amygdalar neurons that are primarily excited by sensory signals, and amygdalar neurons that are excited by both sensory signals and plan-related top-down signals. The latter category of amygdalar neuron must project to sensory TRN to facilitate anticipatory or persistent selective attention when a salient stimulus is not currently present in the environment.

The system can overcome interneuron-mediated attentional resetting if there is a boost to the excitatory input received by BA principal neurons. We model this additional excitation as top-down motivation or bias that excites the plan cortex. This boosts the level of excitation from plan cortex to the BA principal neurons that were previously reinforced, negating the effect of local expectation-related inhibition. Therefore, top-down bias or drive leads to the persistence of attention in the face of both success (expectation confirmation) and failure (expectation violation). Top-down bias effectively reduces the system's sensitivity to expectation-related information.

Topographic mappings between each of the three maps are assumed. Given the differences in the number of neurons in the various interconnected brain regions, it is likely that there will be convergence and divergence of connections between groups of neurons. Conceptually, the activity of each model neuron can also be interpreted as the cumulative activity of a group of neurons with similar connectivity patterns. The key assumption that facilitates selective attention in the model is that each BA neuron projects to roughly the same neurons in the sensory map that project to the LA neurons which excite BA. In other words, the connectivity between individual BA neurons and CRT loops must not be all-to-all. As long as the projections from BA to the sensory map are selective and not uniform, BA neurons that label stimuli with affective salience can select CRT loops that represent the same stimuli. The ‘sharpness’ or ‘resolution’ of BA-dependent selective attention depends on the degree of the spread of the projections from BA to the sensory map.

#### Plan map

The plan map is posited to center on the PFC, which contains several subregions that display activity that correlates with decision-making and planning. For example, ventromedial PFC, which includes OFC, influences several aspects of decision-making, including the integration of emotion- and reinforcement-related information [54]. In humans, lesions of this region lead to serious impairments of decision-making [55,56]. The model plan map represents two examples of behavioral plans that depend on amygdalar input: a fear plan and a feed plan. We assume that there are two model neurons each in plan cortex, TRN, and thalamus, out of which one neuron in each is devoted to the fear plan, and another to the feed plan. We do not assume that these are the only plans for which the amygdala contributes evidence. Appetitive and aversive salience serve as two simple examples of possible valences processed by the amygdala. Physiological studies indicate that neurons in the amygdala fire in response to appetitive and aversive stimuli [24], and therefore ‘feeding’ and ‘fear’ are representative but not exhaustive of plausible behavioral plans or states for which such activity can serve as ‘evidence’. The salience map is presumably constrained by the number of distinct types of affective labeling available in the amygdala. The ability to differentiate among various affective events and label stimuli accordingly depends on a variety of factors including the number of distinct reinforcement- or US-related signals that arrive in the amygdala. Synaptic learning of the type implemented here can allow amygdalar signals and appropriate plans to become bidirectionally linked with each other.

The plan CRT functions as a competitive network. Input from each of the two BA subgroups serves as bottom-up evidence for the corresponding plan. Thus when a stimulus is paired with an aversive US, the corresponding BA principal neuron in the aversive subgroup becomes active, and this in turn activates the fear plan. When a stimulus is paired with an appetitive US, the corresponding BA principal neuron in the appetitive subgroup activates the feed plan. Learning also results in enhancement of the synaptic weights connecting the plan cortex with BA principal neurons. After pairing a neutral stimulus with an aversive US, if there is top-down excitation of the fear plan CRT, then attention will be restricted to the stimulus that corresponds with that plan, i.e., the stimulus previously paired with the aversive US. All other stimuli are suppressed, including stimuli that were paired with the appetitive US. Note that the model pertains to plan activation, but not plan execution.

As in the amygdala, local inhibitory interneurons in plan cortex facilitate resetting in the event of expectation confirmation and expectation violation (Fig 1, Inset F). The activity of the plan cortical neuron represents an expectation that the plan will eventually lead to the corresponding US. Thus overlap between plan cortical activity and the corresponding US constitutes a confirmation of expectation. Activation of each plan cortical neuron triggers the gradual build-up of an expectation violation signal, which prevents the system from getting stuck ‘waiting’ for an affective prediction to be validated. Top-down biasing of the plan cortex allows the plan cortical neuron to overcome the resetting effect of the inhibitory interneuron, facilitating fixed, focused attention that is not ‘discouraged’ by prediction failure. As in the amygdala, expectation confirmation counteracts the build-up of the expectation violation signal.

The amygdala and the plan cortex represent two different levels of expectation. In the amygdala, the expectation of US delivery is stimulus-specific, whereas in the plan cortex, the expectation is plan-specific. The plan-specific expectation is more general: it represents the prediction that some stimulus will be reinforced, but not a particular stimulus. The top-down excitation from the plan cortex to BA allows the plan to excite all stimuli in the sensory map that were previously associated with the corresponding US. In other words, the pathway from plan cortex to BA to the sensory map facilitates a form of relevance detection [57]. Stimuli that are consistent with the currently active plan are attended to, and irrelevant stimuli are suppressed, even if they had been labeled as salient with a different US.

## Results

We simulated two variants of the EmGate model: a rate-coded model, and a spiking model based on Izhikevich neurons [58]. The results for the two variants were very similar, which indicates that the key properties of the model are robust consequences of the connectivity pattern. Figures demonstrating the overall behavior of the model were derived from the rate-coded simulations. Since the spiking simulations reveal no new qualitative behavior, we used the spiking results to focus on a smaller temporal window, which allows clear visualization of the contribution of inhibitory interneurons to flexibility.

**Fig 2:**
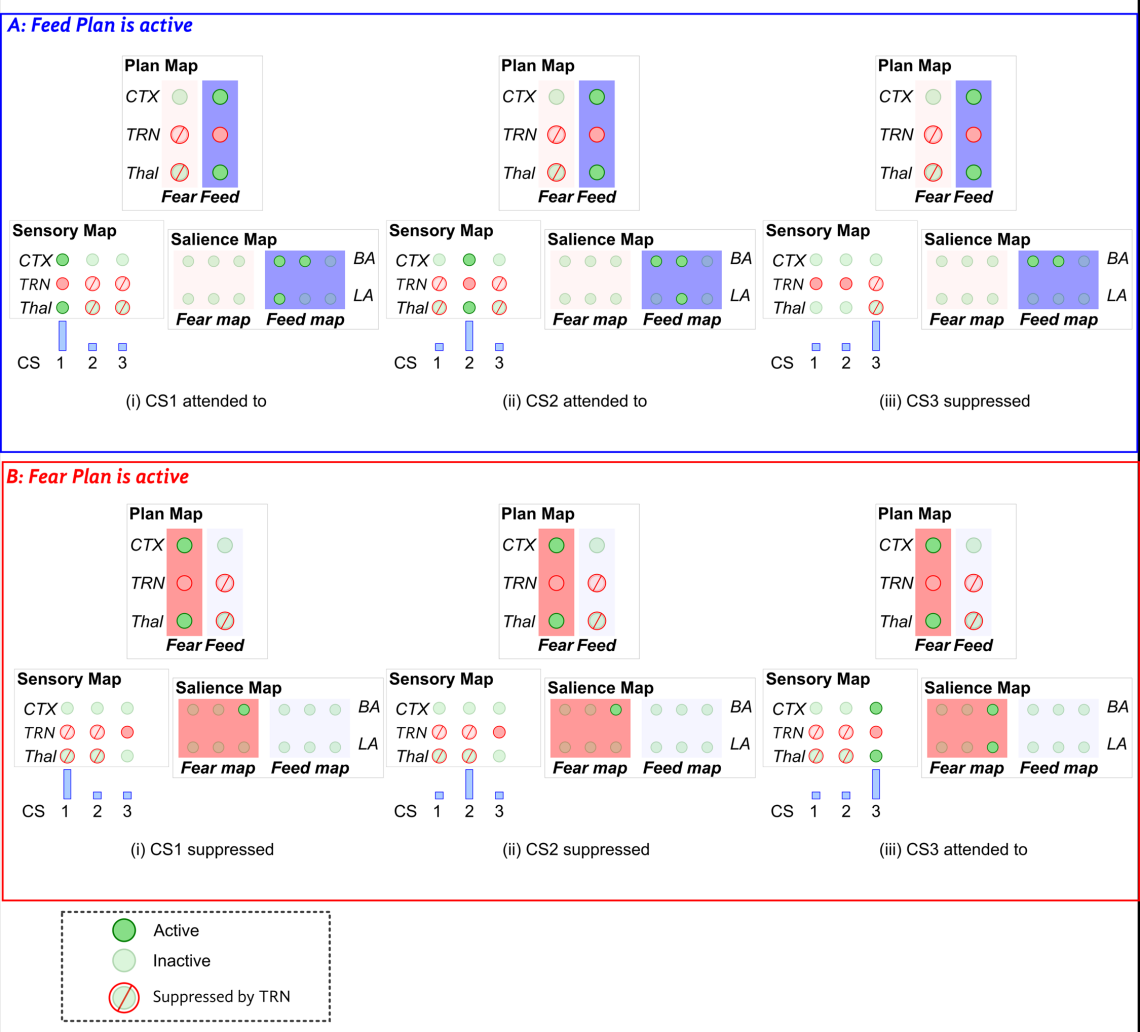
Attentional suppression and selection by the EmGate model. ***A*,** When the feed plan is active, only CS1 and CS2 can pass through the attentional gate (i) and (ii), but CS3 is suppressed via the BA-TRN projection (iii). ***B*,** When the fear plan is active, CS1 and CS2 are suppressed (i), (ii) and CS3 is attended to (iii). Note that LA neurons are only active when the corresponding CS is present [A(i), A(ii), B(iii)]. By contrast, BA neurons receive excitation from plan cortex, which allows the BA-TRN projection to mediate suppression of distractors and irrelevant stimuli even when the corresponding CS is not present. The subplots on the left show the behavior of the model when CS1 is presented (i). The subplots in the center show the behavior of the model when CS2 is presented (ii). The subplots on the right show the behavior of the model when CS3 is presented (iii).

We studied the performance of the EmGate model for two emotional attention paradigms: a Pavlovian conditioning experiment and an ‘attentional rubbernecking’ experiment. For the Pavlovian simulations, each simulation run is divided into four epochs or phases: two reinforcement epochs followed by two testing phases. During the first epoch, two conditioned stimuli (CS1 and CS2) are associated with appetitive reinforcement, such as food. During the second epoch, a third conditioned stimulus (CS3) is associated with aversive reinforcement, such as an airpuff. Thus, there is a distinct US for each type of reinforcement. During the testing phases, CS1, CS2 and CS3 are presented multiple times sequentially. Additional neutral distractor stimuli are included during the testing phases. During the second testing phase, top-down motivation or bias is added to one of the two plans. Each conditioned stimulus is presented in isolation and is separated from the subsequent stimulus by an inter-stimulus interval. Thus, there is no temporal overlap between CS1, CS2 and CS3. The basic functioning of the model is depicted schematically in Fig 2. (Details of behavioral paradigms and model simulations are provided in Methods.)

### Bottom-up shifting of attention tracks valence of stimuli and contributes to deciding on a behavioral plan

**Fig 3:**
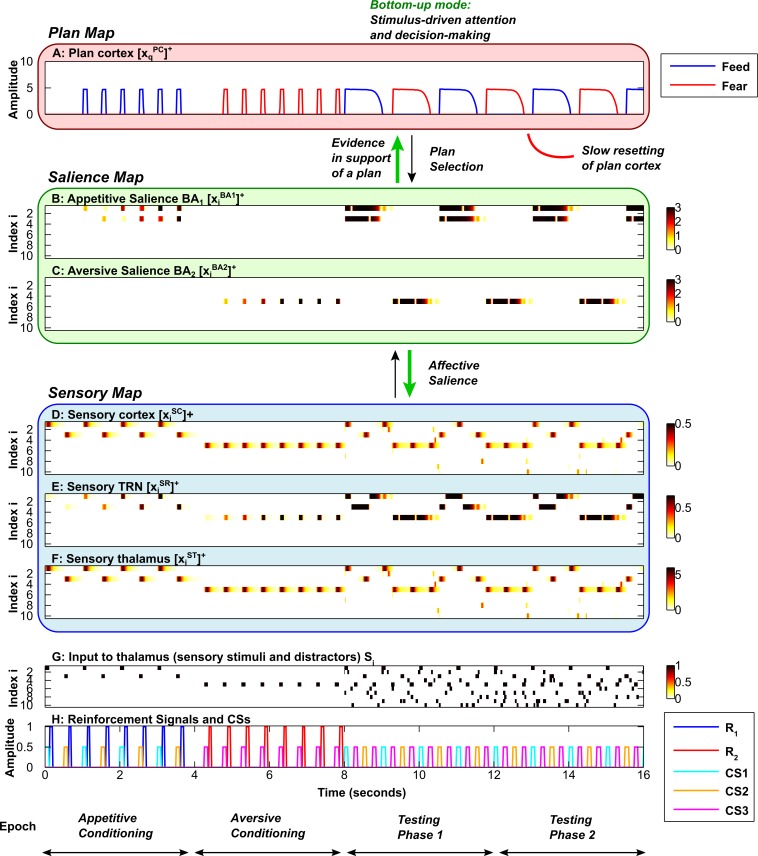
Slow flexible modulation of attention by emotion. Each simulation is divided into 4 epochs (bottom): two conditioning phases, followed by two testing phases. Each subplot shows the time evolution of key model activities. ***A*,** Plan map. ***B*,** Salience map for appetitive (positive) stimuli. ***C*,** Salience map for aversive (negative) stimuli. ***D*, *E*, *F*,** Sensory map, including sensory cortex (D), sensory TRN sector (E) and sensory thalamus (F). ***G*,** Input to sensory thalamus (stimuli and distractors). ***H*,** Reinforcement signals and Conditioned Stimuli (CSs). Amplitudes of CSs are scaled down to 0.5 for visibility (H). In B-G, the y-axis represents the neuron index (neuron #); white indicates zero activity, and dark red/black indicates high activity. In A and H, the y-axis represents activity. In all subplots the x-axis represents time. During the first conditioning phase, CS1 and CS2 are associated with a food reward. During the second conditioning phase, CS3 is associated with an aversive stimulus. During both testing phases, the conditioned stimuli are presented repeatedly in the sequence CS1-CS3-CS2-CS3, with no reinforcement. Elevated activity of a sensory cortical neuron (D) corresponds to attention allocated to the corresponding stimulus (H). Elevated activity in an amygdalar BA principal neuron in a given subpopulation (B and C) represents the affective salience of the corresponding stimulus (H). Elevated activity in a plan cortical neuron (A) indicates that the corresponding behavioral plan is activated. During the testing phases, the rate at which the plan (A) shifts from feed (blue) to fear (red) is dependent on the rate of build-up of the plan cortical expectation violation (EV) signal. Here the cortical expectation violation signal builds up slowly in comparison with Fig 4, allowing each plan (A) to persist for some time despite stimulus-specific violations of expectation. In the bottom-up mode (green arrows), which depends on external stimuli, processing in the salience map (B, C) can (1) determine affective salience, focusing and shifting attention in the sensory map (D-F), and (2) provide evidence in support of plans corresponding to appetitive or aversive stimuli, driving decision-making and switching between the feed (blue plot) and fear (red plot) plan (A).

**Fig 4:**
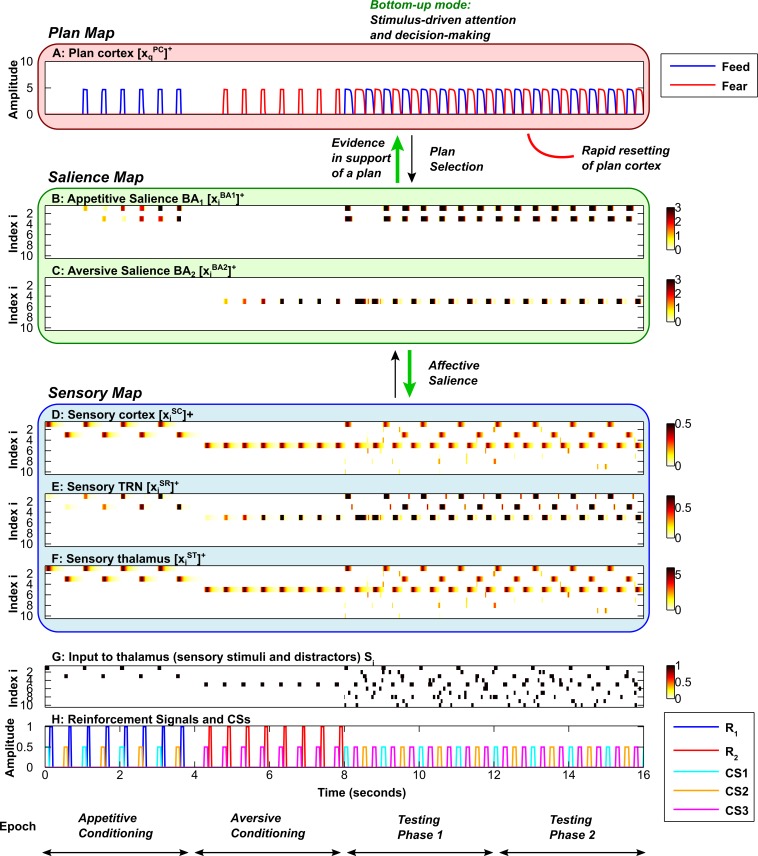
Rapid flexible modulation of attention by emotion. Axis and subplot labels are as in Fig 3. ***A*,** Plan map. ***B*,** Salience map for appetitive (positive) stimuli. ***C*,** Salience map for aversive (negative) stimuli. ***D*, *E*, *F*,** Sensory map, including sensory cortex (D), sensory TRN sector (E) and sensory thalamus (F). ***G*,** Input to sensory thalamus (stimuli and distractors). ***H*,** Reinforcement signals and Conditioned Stimuli (CSs). During the testing phases, the rate at which the plan (A) shifts from feed (blue) to fear (red) is dependent on the rate of build-up of the plan cortical expectation violation (EV) signal. Here the cortical expectation violation (EV) signal builds up rapidly in comparison with Fig 3, causing the plan to shift after a single expectation violation (A). The speed of resetting of cortical plan representations (A) determines how often there is a temporal window of opportunity for bottom-up emotion-related signals (green arrows, B and C) to determine plans (A) and sensory attention (D, F).

The activities of the sensory cortical and thalamic neurons in the EmGate model represent sensory attention (Figs 3D, 3F, 4D and 4F). When reinforcement is delivered after a stimulus, rapid synaptic learning occurs in the amygdala (Figs 3B, 3C, 4B and 4C). The process of amygdalar ‘labeling’ of stimuli directs attention to the labeled stimulus. Accompanying this boost in attention is the activation of a plan (Fig 3A and Fig 4A) that corresponds to the nature of reinforcement (Fig 3H and Fig 4H). In the case of food reward, a feed plan is activated. In the case of airpuffs, a fear plan is activated. Attention is restricted to stimuli that are relevant to the active plan. When resetting of the cortical plan neuron occurs, the system’s sensitivity to bottom-up salience signals from the amygdala is restored. Resetting allows the system to shift attention to the most salient stimulus currently present, and choose the corresponding behavioral plan (Fig 3A–3C and Fig 4A–4C, testing phases). Thus, following the resetting of a plan, the system can reassess the bottom-up evidence provided by the salience map and change course if the situation demands it. Resetting can be relatively slow (Fig 3A–3C), or it can occur fast enough for plans and attention to shift after a single stimulus is presented (Fig 4A–4C).

Each US triggers a corresponding reinforcement signal, which allows for long-term potentiation (LTP) of the synaptic connections between sensory thalamus and LA, and between plan cortex and amygdala. Distractors are suppressed, except when they occur during the brief transition periods when low amygdalar activity leads to weakened TRN inhibition of non-salient stimuli. Thus the amygdala-TRN projection restricts thalamo-cortical signaling so that only salient signals reach the cortex.

### Top-down bias focuses attention through suppression of irrelevant stimuli

**Fig 5:**
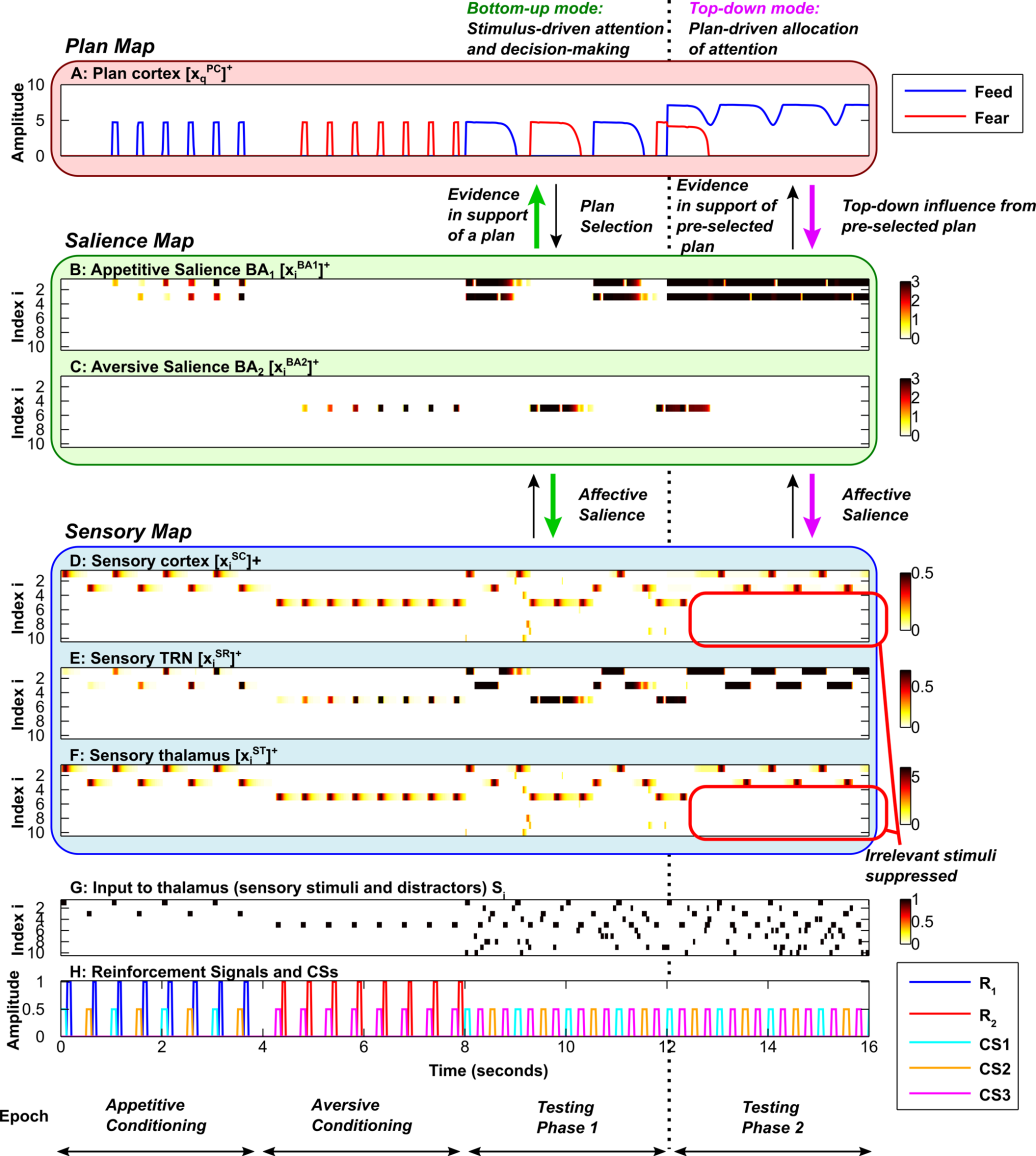
Focused top-down attention on food-related stimuli. Axis and subplot labels are as in Fig 3. ***A*,** Plan map. ***B*,** Salience map for appetitive (positive) stimuli. ***C*,** Salience map for aversive (negative) stimuli. ***D*, *E*, *F*,** Sensory map, including sensory cortex (D), sensory TRN sector (E) and sensory thalamus (F). ***G*,** Input to sensory thalamus (stimuli and distractors). ***H*,** Reinforcement signals and Conditioned Stimuli (CSs). The simulations reveal that the amygdala-TRN projection can mediate both bottom-up (green arrows) and top-down (purple arrows) modulation of attention by emotion. During the first conditioning phase, CS1 and CS2 are associated with a food reward. During the second conditioning phase, CS3 is associated with an aversive stimulus. During the first testing phase (similar to Fig 3), the conditioned stimuli are presented repeatedly in the sequence CS1-CS3-CS2-CS3, with no reinforcement (bottom-up mode, green arrows). During testing phase 1, the rate at which the plan (A) shifts from feed (blue) to fear (red) is dependent on the rate of build-up of the plan cortical expectation violation (EV) signal. Here the cortical expectation violation signal builds up slowly (similar to Fig 3), allowing each plan (A) to persist for some time despite stimulus-specific violations of expectation. However, during the second testing phase (to the right of the vertical dotted line) top-down bias is applied to the feed plan (A–blue plot). This allows the feed plan to overcome the effects of expectation violation (EV), and remain active (in this simulation *M*_*1*_ = 160 during the second testing phase). Top-down excitation of BA neurons (purple arrow) allows BA (B) to direct attention only to the stimuli that were previously associated with food: CS1 and CS2. Attention to CS3 (D, F) is attenuated, despite the fact that it has been labeled as salient by virtue of its association with an aversive stimulus. Top-down cortical biasing (A) of BA can restrict attention to a particular category of affectively salient stimuli without restricting attention to only one stimulus. In other words, the system can divide attention between CS1 and CS2. Thus, even when the plan itself is locked in place by the top-down bias, there is within-plan flexibility to shift sensory attention. This flexibility arises because expectation violation signals cause inhibition of BA neurons.

**Fig 6:**
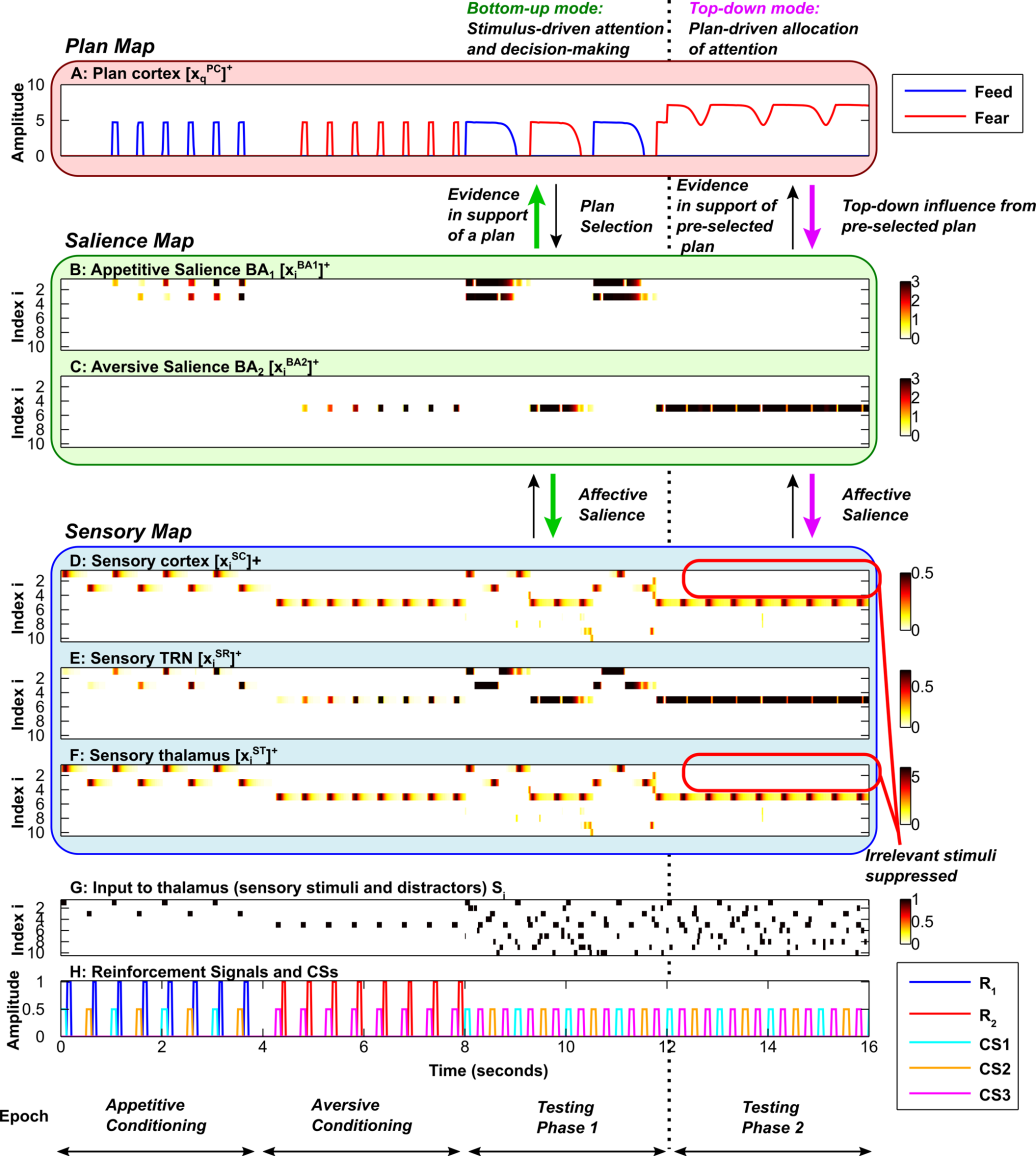
Focused top-down attention on fear-related stimuli. Axis and subplot labels are as in Fig 5. ***A*,** Plan map. ***B*,** Salience map for appetitive (positive) stimuli. ***C*,** Salience map for aversive (negative) stimuli. ***D*, *E*, *F*,** Sensory map, including sensory cortex (D), sensory TRN sector (E) and sensory thalamus (F). ***G*,** Input to sensory thalamus (stimuli and distractors). ***H*,** Reinforcement signals and Conditioned Stimuli (CSs). The simulations reveal that the amygdala-TRN projection can mediate both bottom-up (green arrows) and top-down (purple arrows) modulation of attention by emotion. During the second testing phase (to the right of the vertical dotted line) top-down bias is applied to the fear plan. This allows the fear plan (A–red plot) to overcome the effects of inhibition elicited by expectation violation (EV) and remain active (in this simulation *M*_*2*_ = 160 during the second testing phase). Top-down excitation of BA neurons (purple arrows) allows BA (C) to restrict sensory attention to the stimulus that was previously associated with an aversive stimulus: CS3. Attention to CS1 and CS2 is attenuated (D, F), despite the fact that they have been labeled as salient.

Motivation or bias towards food could result from extreme hunger and aversive bias could result from heightened fear. In the model, the bias provides additional excitation to one of the two behavioral plans. This in turn causes an attentional bias mediated by the projection from the amygdala to the sensory TRN, restricting attention to the stimuli previously paired with the reinforcement that corresponds to the chosen plan and blocking stimuli that are irrelevant. If the model is biased towards feeding, it restricts attention to the two stimuli that were paired with food and ignores the stimulus previously paired with airpuffs (Fig 5). If the model is instead biased towards fear, it restricts attention to the stimulus that was paired with airpuffs, and ignores the two stimuli that were associated with food (Fig 6). Therefore, when there is top-down bias or motivation, stimuli that are not relevant to the pre-selected plan are actively suppressed and therefore ‘hidden’ from cortex, even if they are salient. In the simulations, top-down bias is activated during Testing Phase 2 (Figs 5 and 6, purple arrows, right of vertical dotted line).

### Local cortical inhibition allows flexible decision-making

**Fig 7:**
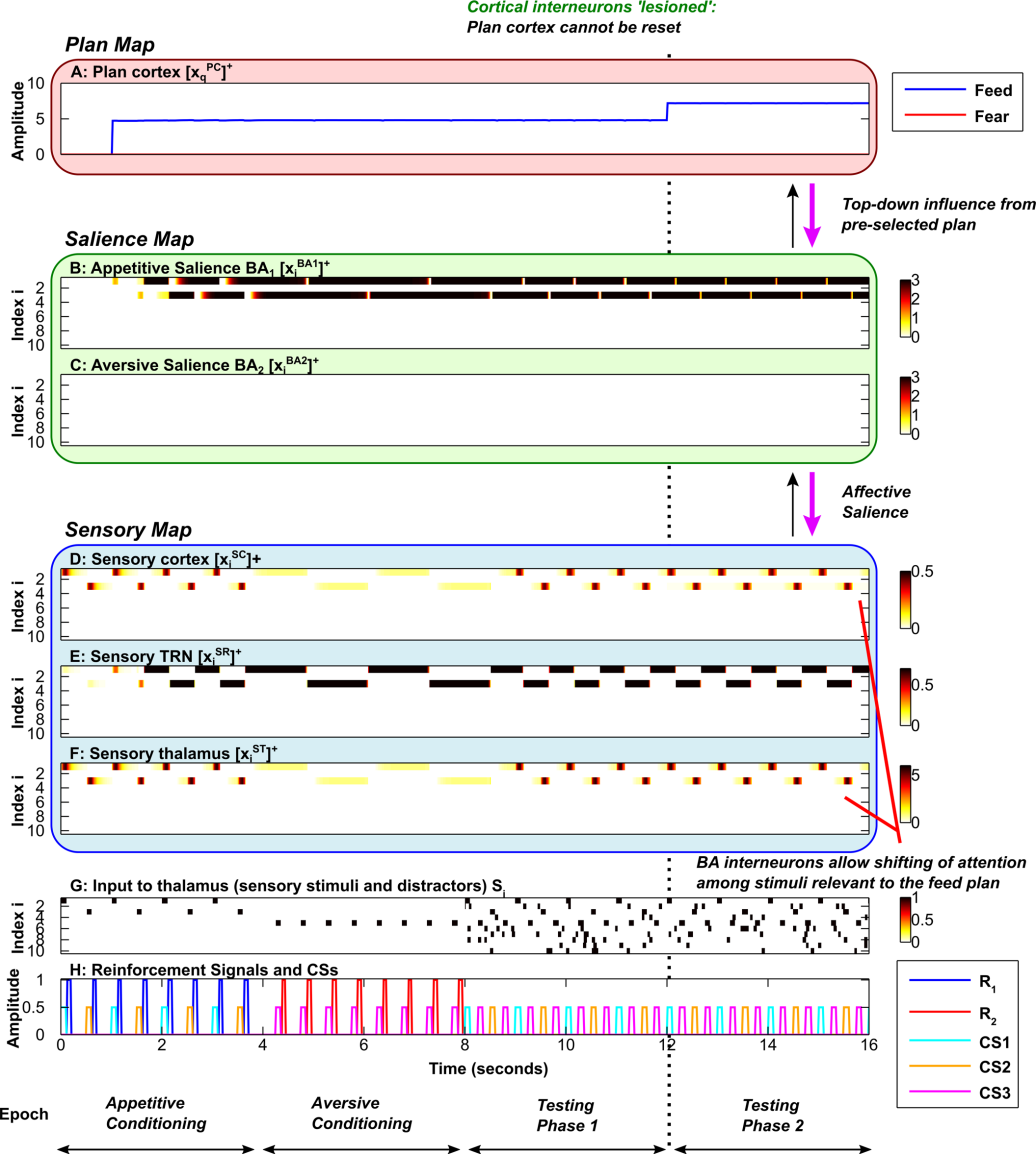
Impaired decision-making flexibility in the absense of inhibitory interneurons in plan cortex. Axis and subplot labels are as in Fig 5. ***A*,** Plan map. The plan cortical inhibitory interneurons are 'lesioned' in the simulation. ***B*,** Salience map for appetitive (positive) stimuli. ***C*,** Salience map for aversive (negative) stimuli. ***D*, *E*, *F*,** Sensory map, including sensory cortex (D), sensory TRN sector (E) and sensory thalamus (F). ***G*,** Input to sensory thalamus (stimuli and distractors). ***H*,** Reinforcement signals and Conditioned Stimuli (CSs). During the second testing phase (to the right of the vertical dotted line) top-down bias is applied to the feed plan. The plans (A) cannot shift as in the previous simulations. Thus in the absense of inhibition onto the plan cortical neurons (triggered by expectation confirmation and violation), the decision-making flexibility of the system is degraded. (In this simulation *M*_*1*_ = 160 during the second testing phase).

Inhibitory interneurons in the plan cortex serve a crucial role in resetting the competitive decision-making process. Each interneuron interrupts the excitatory feedback loop between thalamic and cortical neurons that correspond to the same plan, creating a window of opportunity for new affective information from the amygala to contribute to a shift in the behavioral plan. Each interneuron is excited by expectation confirmation as well as expectation violation (Fig 1, Inset F). Expectation confirmation occurs when plan cortical activity co-occurs with the corresponding US. If a food plan is activated, then the arrival of food triggers inhibition of the plan, allowing the system to move on to a different plan. Expectation confirmation is insufficient to confer adequate flexibility in realistic situations, however. Expectations and predictions frequently fail, and it is often inappropriate to persist with a plan in the face of such failures. For this reason an expectation violation signal must also be present for each plan, facilitating the eventual inhibition of a plan that has not resulted in the expected outcome. The rate at which the expectation violation signal builds up determines how quickly the system resets itself and becomes open to new salient information from the amygdala. A slow build-up of expectation violation results in a more ‘patient’ decision-making process that allows the system to persist with a plan for some time before ‘giving up’. A rapid build up of expectation violation results in an ‘impatient’ decision-making process that quickly shifts in the face of prediction failure. Resetting the competition between behavioral plans is only possible in the bottom-up mode, when there is no extrinsic bias or motivation. The ability of the plan cortical interneurons to bring about this resetting is counteracted by extrinsic excitatory drive in the top-down mode, which allows the system to persist with a chosen plan indefinitely. A similar persistence of behavioral plan can be induced by ‘lesioning’ the plan cortical interneurons (Fig 7). Without plan cortical interneurons, the system is unable to inhibit the ongoing plan, resulting in inappropriately rigid behavior and a failure to detect and learn the salience of new stimuli (Fig 7). Thus, plan cortical interneurons contribute not only to flexible decision-making, but also indirectly to the ability to learn new affective associations. However, the flexibility of the system is not completely destroyed by lesioning the cortical interneurons. Without cortical interneurons, the system is still able to learn that multiple stimuli (CS1 and CS2) are associated with the feed plan, and to shift attention among these stimuli (Fig 7B). This residual ability to learn and to shift attention is present because the amygdalar interneurons, discussed below, are intact.

### Local amygdalar inhibition allows flexible shifting of attention

**Fig 8:**
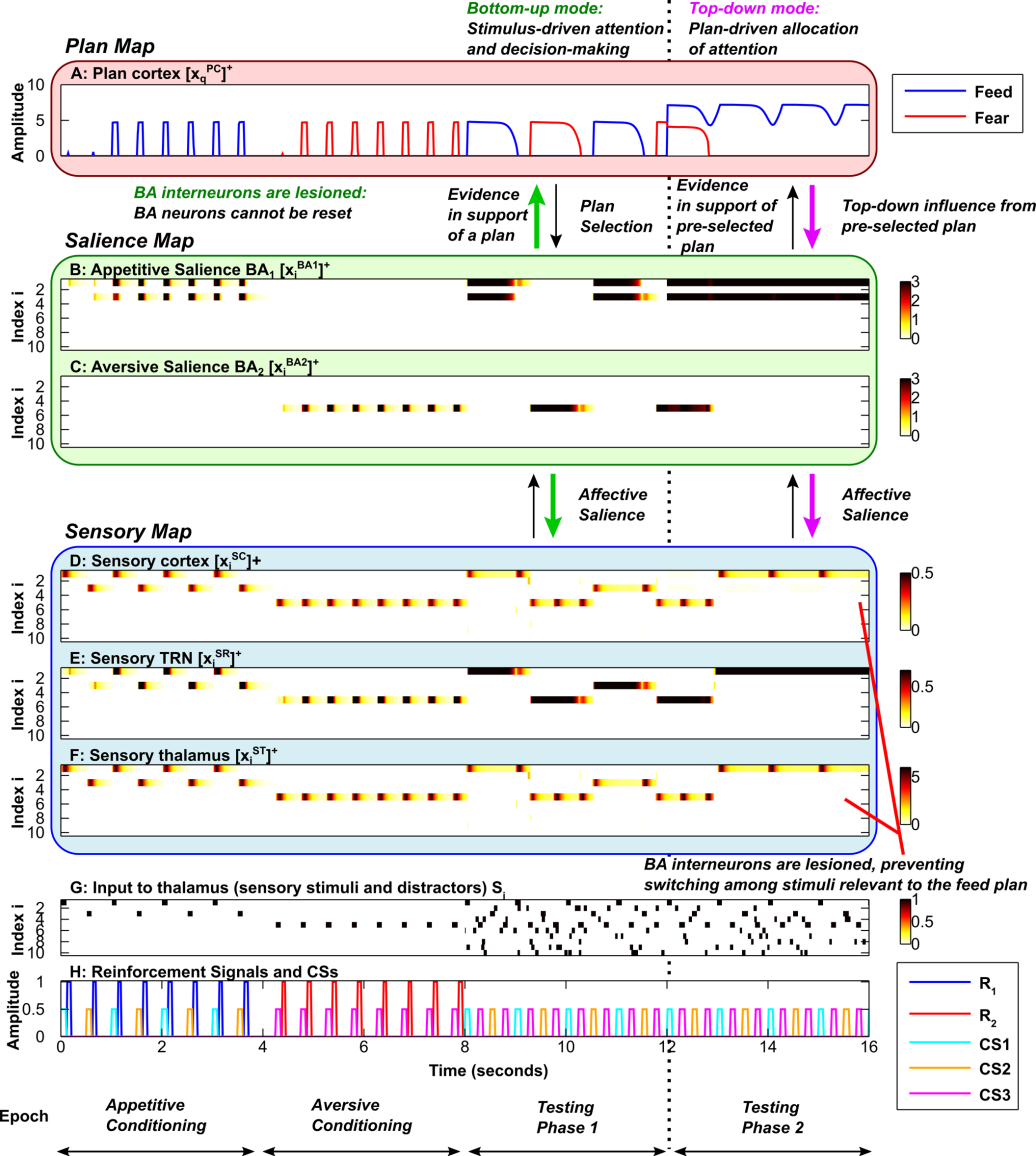
Attentional flexibility is impaired in the absense of BA inhibitory interneurons. Axis and subplot labels are as in Fig 5. ***A*,** Plan map. ***B*, *C*,** Salience map. The BA interneurons are ‘lesioned’ in the simulation. ***D*, *E*, *F*,** Sensory map, including sensory cortex (D), sensory TRN sector (E) and sensory thalamus (F). ***G*,** Input to sensory thalamus (stimuli and distractors). ***H*,** Reinforcement signals and Conditioned Stimuli (CSs). During the second testing phase (to the right of the vertical dotted line) top-down bias is applied to the feed plan. The plans (A) shift as in the previous simulations, but within the periods when the feed plan (blue plot) is active, sensory attention (D) does not shift between the two relevant stimuli, CS1 and CS2. Attentional flexibility of the system is thus degraded in the absense of inhibition onto the BA neurons (triggered by stimulus-specific expectation violation). During the first testing phase, the rate at which the plan shifts from feed to fear is dependent on the rate of build-up of the cortical expectation violation signal. A slow build-up is employed, as in Fig 3. (In this simulation *M*_*1*_ = 160 during the second testing phase).

**Fig 9:**
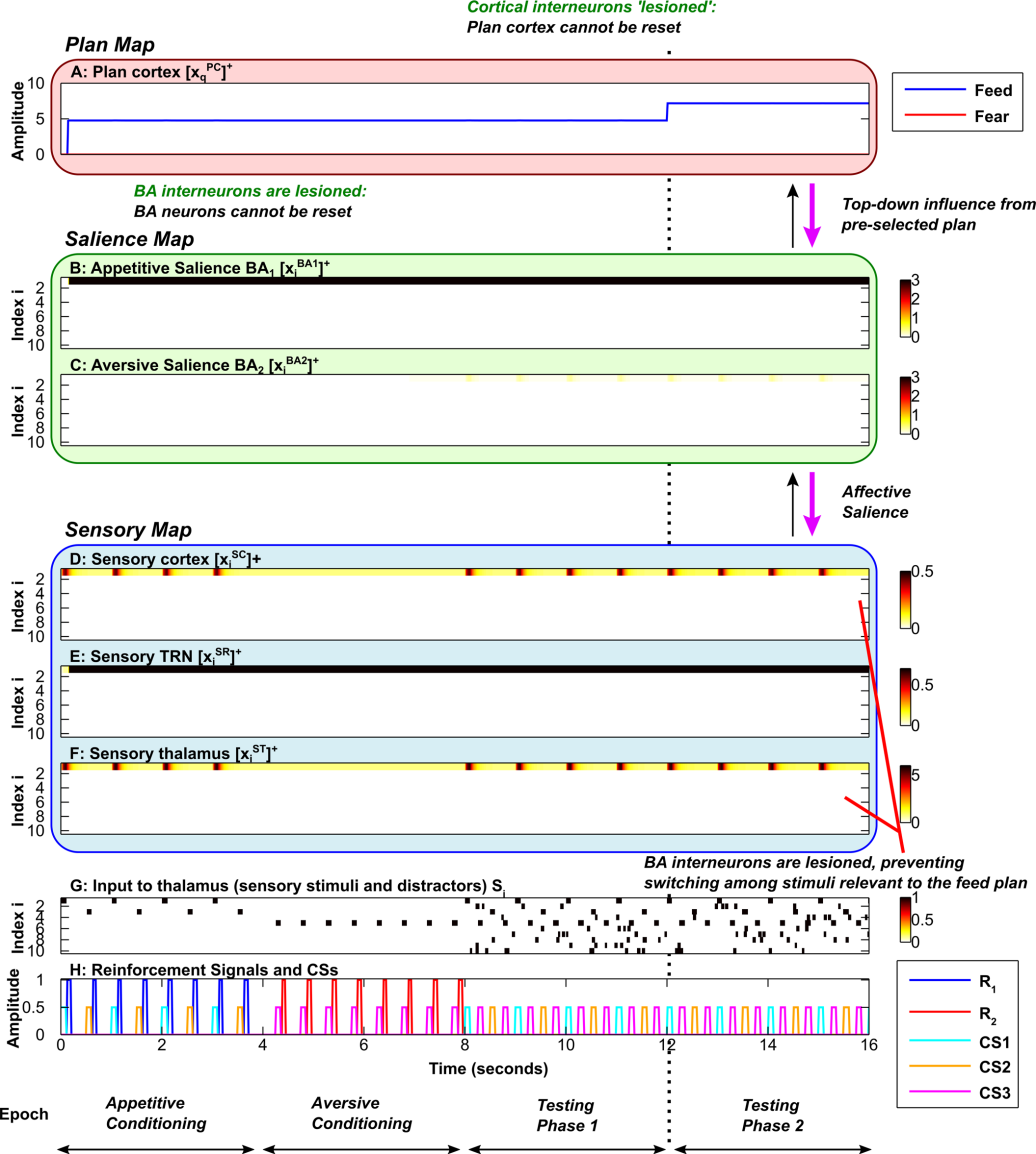
Inflexibility in the absense of both BA interneurons and plan cortical interneurons. Axis and subplot labels are as in Fig 5. ***A*,** Plan map. The plan cortical interneurons are ‘lesioned’ in the simulation. ***B*, *C*,** Salience map. The BA interneurons are ‘lesioned’ in the simulation. ***D*, *E*, *F*,** Sensory map, including sensory cortex (D), sensory TRN sector (E) and sensory thalamus (F). ***G*,** Input to sensory thalamus (stimuli and distractors). ***H*,** Reinforcement signals and Conditioned Stimuli (CSs). When the interneurons in BA and plan cortex are 'lesioned' the simulation reveals inability of the network to reset the cortical plan signals (A) as well as the sensory attentional modulation from BA (B, C), leading to pathological inflexibility in both attention (D, F) and decision-making (A). During the second testing phase (to the right of the vertical dotted line) top-down bias is applied to the feed plan. (In this simulation *M*_*1*_ = 160 during the second testing phase).

**Fig 10:**
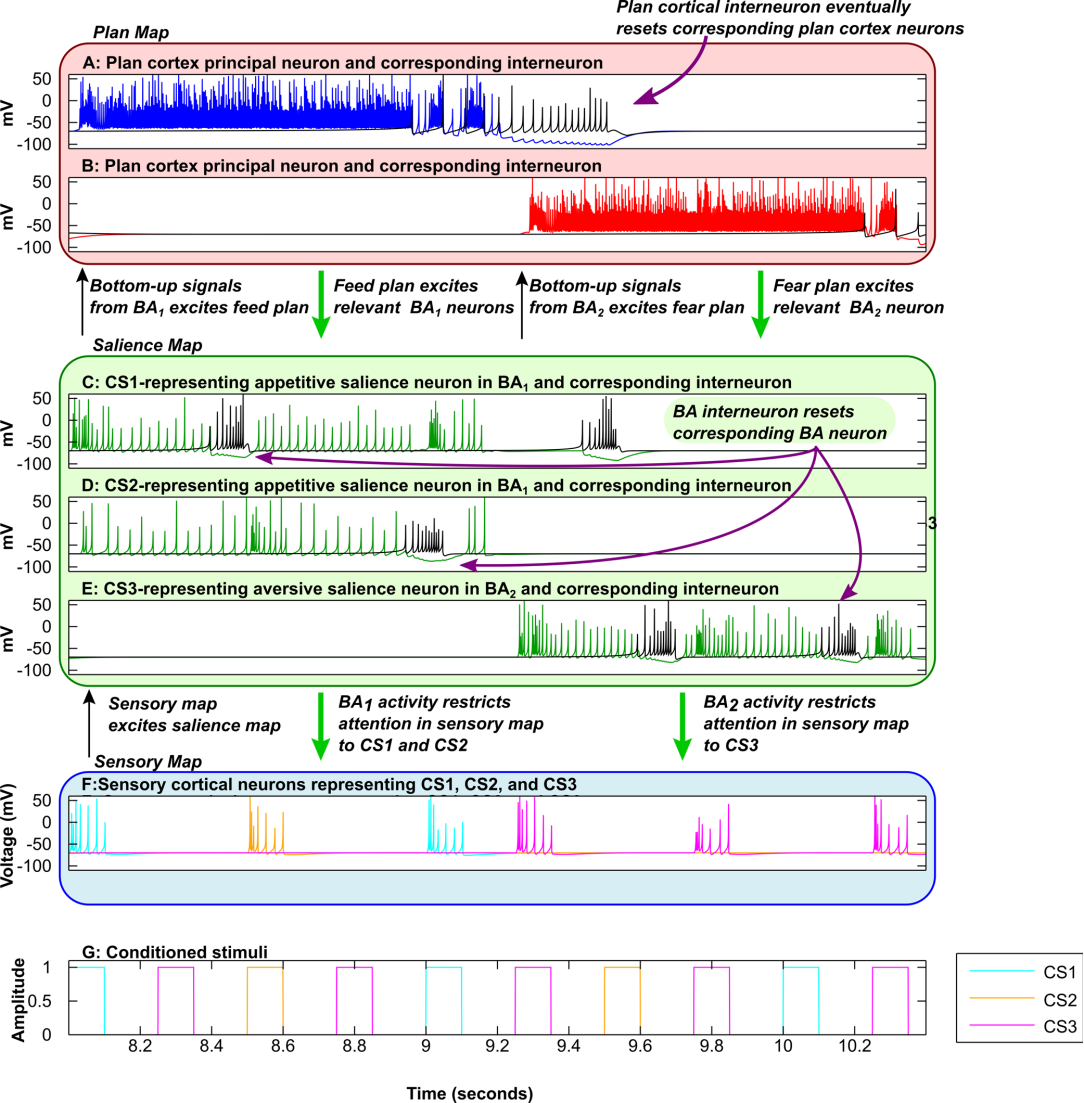
Contributions of cortical and amygdalar interneurons to flexible decision-making and attention: Spiking model. Time evolution of a key spiking model activity, during the first two seconds of the first testing phase. Simulations and key activities of the rate-coded and spiking models were similar, with each simulation divided into 4 epochs: two conditioning phases, followed by two testing phases. ***A*,** Cortical activity corresponding to the feed plan. ***B*,** Cortical activity corresponding to the fear plan. Plan cortical inhibitory interneurons (black spikes) trigger resetting of the corresponding plan (blue and red spikes), allowing a new plan to be selected. ***C*, *D*,** Activity in the salience map corresponding to appetitive (positive) stimuli (CS1, CS2). ***E*,** Activity in the salience map corresponding to an aversive (negative) stimulus (CS3). Amygdalar inhibitory interneurons (black spikes) trigger resetting of the corresponding BA principal neuron (green spikes), allowing a new CS to be selected if it is relevant to the ongoing plan (C-E). ***F*,** Activity in sensory cortex corresponding to CS1 (cyan), CS2 (orange), and CS3 (magenta). ***G*,** Conditioned Stimuli reaching sensory thalamus (CSs). In A-E, the inhibitory interneuron spiking activity is shown in black.

When a particular plan is activated, the system is capable of shifting sensory attention among stimuli that are relevant to that plan. This is because there is stimulus-specific inhibition in the amygdala that complements the plan-specific inhibition in the plan cortex. As in the plan cortex, both expectation confirmation and expectation violation signals excite the local inhibitory interneurons. The expectations represented in the salience map operate on a more fine-grained level than those in the plan map. While the plan map represents the expectation that the plan will be followed by the corresponding US, the salience map represents the expectation that a particular stimulus will be followed by the corresponding US. In the absence of amygdalar interneurons, the system can still shift plans and attend to salient stimuli. However, the ability to shift sensory attention among stimuli relevant to the same plan is degrated. Thus, when amygdalar interneurons are ‘lesioned’, sensory attention is assigned to the first relevant stimulus that arrives, but cannot shift to other relevant stimuli (Fig 8). If both cortical and amygdalar interneurons are ‘lesioned’, both behavioral plan and sensory attention become pathologically inflexible (Fig 9). The contributions of plan cortical and amygdalar interneurons are shown in Fig 10, which zooms in on the first six stimulus presentations in the first testing phase. The figure is drawn from simulations of the spiking variant of the EmGate model.

### Inhibition of thalamus via amygdala-TRN pathway as a mechanism for emotion-induced blindness

**Fig 11:**
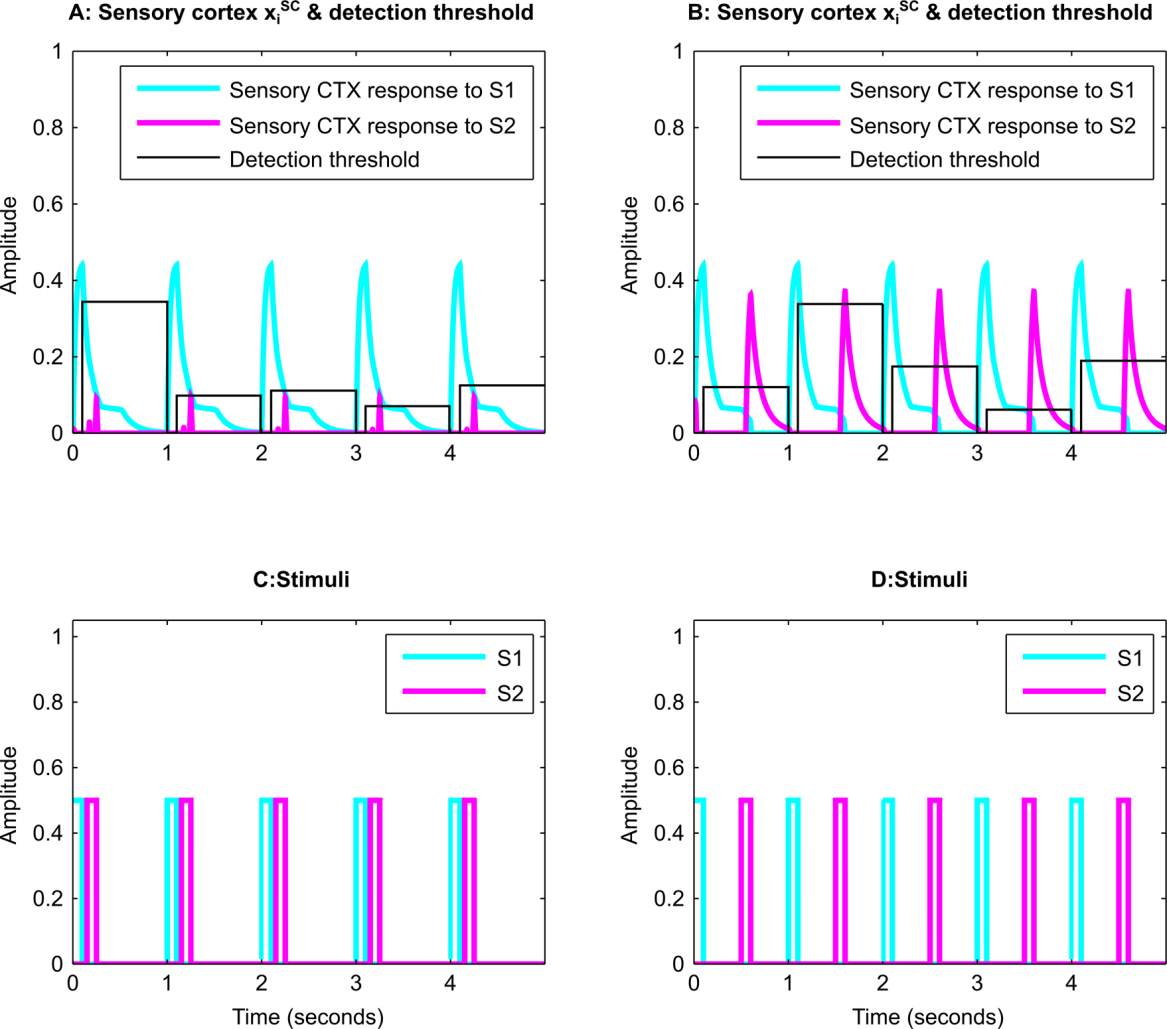
‘Emotion-induced blindness’. ***A*, *B*,** Time evolution of the activity of the sensory cortical neurons corresponding to the target stimulus S1 (blue lines) and to the target stimulus S2 (pink lines) in the short lag (C) or long lag (D) condition. ***C*, *D*,** Presence of stimuli S1 (blue) and S2 (pink), separated by a short (A) or long (B) lag. The stimulus S1 was previously paired with an aversive US. If the time lag between the salient stimulus S1 and the target stimulus S2 is short (A, C), S1 causes suppression of representations of S2 via the amygdala-TRN pathway. If the time lag between the salient stimulus S1 and the target stimulus S2 is long (B, D), the inhibition triggered by S1 does not persist long enough to suppress S2. A stimulus is assumed to be detected if its cortical representation (A, B) crosses the variable detection threshold (black lines). Five detection trials out of a total of twenty are shown. The detection success rate for the short lag was 10%, and for the long lag was 100%. In all subplots the x-axis represents time and the y-axis represents amplitude.

We also simulated a simplified version of the ‘attentional rubbernecking’ phenomenon [4], in which an affective stimulus leads to weakened ability to detect subsequent neutral stimuli if the lag between stimuli is small. In our simulation of this paradigm, two stimuli (S1 and S2) are presented one by one, separated by a time lag (Fig 11C and 11D). The task involves detection of the second, neutral stimulus. Successful detection is assumed to occur when the sensory cortical activity corresponding to the neutral stimulus crosses a threshold (Fig 11A and 11B). If S1 has previously been paired with a US, the accuracy of detection of S2 is reduced when the time lag is short (Fig 11A and 11C), but not if the time lag is long (Fig 11B and 11D). Stimulus-induced blindness depends on the emotional salience of S1. If S1 is a neutral stimulus there is no reduction in detection accuracy at any lag. In contrast, an affectively salient stimulus ‘lingers’ in the emotional salience map compared to a neutral stimulus, and therefore continues to affect activity in the sensory map and the plan map even after the stimulus is turned off. A stimulus will be occluded if it arrives before the amygdalar interneurons can shut off the activity of principal neurons that project to TRN. The model demonstrates this behavior because of the net inhibitory effect of the amygdala-TRN pathway on non-salient stimuli. Our results are consistent with the experimental findings from the attentional rubbernecking paradigm [4].

## Discussion

The EmGate model demonstrates four computational processes mediated by the circuitry linking amygdala, TRN and cortex: (1) bottom-up shifting of both attention and behavioral plan in response to the affective salience of the presented stimulus; (2) top-down focusing of attention on stimuli that are relevant to a pre-selected behavioral plan, accompanied by suppression of irrelevant stimuli; (3) flexible resetting of decision-making via inhibitory inteneurons in the cortex and (4) flexible shifting of attention via inhibitory interneurons in the amygdala. The model also demonstrates how these mechanisms can provide a parsimonious explanation of the ‘emotion-induced blindness’ phenomenon [4]. These results support the idea that the recently discovered pathway from amygdala to TRN contributes to cognitive-emotional interactions [5]. The model suggests a mechanism that can account for the correlation between neural activity in the primate amygdala and attention to positive and negative stimuli [25]. The results demonstrate that the interactions between the amygdala and prefrontal cortex are not necessarily antagonistic. Instead, the connectivity allows for powerful top-down and bottom-up forms of cooperative interaction between cortex and amygdala. To our knowledge the EmGate model is the first computational model to explore the functional implications of the amygdala-TRN pathway.

### Inhibition as a key facilitator of selectivity and flexibility

The EmGate model demonstrates how flexible affective attention and decision-making can be mediated by a circuit linking the amygdala with cortico-reticulo-thalamic loops. Inhibitory mechanisms prove to be crucial in enabling the two key properties of the model: selectivity and flexibility. The inhibitory projections from TRN mediate selectivity, whereas the inhibitory interneurons in cortex and amygdala mediate flexibility.

Our simulations highlight the distinctive status of the projections from the amygdala to the sensory sectors of TRN. The primary source of excitation to the sensory map is from external stimuli. In the EmGate model, the pathway from the amygdala to the sensory sectors of TRN powerfully suppresses thalamic activity that is either unimportant or irrelevant to the ongoing plan. The ability of the amygdala-TRN projection to restrict attention to salient stimuli that are pertinent to a pre-selected plan suggests a powerful mechanism by which the amygdala can serve as a relevance-detection system. Such a role has been proposed as a unifying explanation of a variety of neuroimaging and patient studies of the human amygdala, and as an alternative to the idea that the amygdala serves as a dedicated ‘fear module’ [57]. The extent of the amygdala’s ability to suppress non-salient and irrelevant stimuli ultimately depends on the extent of off-surround inhibition of the thalamus by the TRN. Cross-modal suppression would require that neurons in one TRN sector inhibit thalamic neurons representing a distinct modality. In the EmGate model we employ uniform off-surround inhibition, which can be used for selection not only in topographic sensory maps, but also in more abstract categorical spaces. The amygdala-TRN projection is not limited to the sensory domain, but can also contribute to attention and relevance-detection in more complex cognitive domains.

The projection from the amygdala to the sensory TRN serves as a mechanism for emotion-guided inhibitory selection—the very process of selecting a salient stimulus leads to suppression of other stimuli. Such a mechanism can be contrasted with the excitation-based attentional modulation made possible by direct projections from the amygdala to principal neurons in thalamus and cortex [29]. Excitatory attentional mechanisms can at best amplify existing signals in CRT loops, but cannot directly suppress them. As an inhibitory regulator of the thalamus, the TRN is uniquely positioned to preemptively select information for subsequent processing stages including decision-making. The amygdala-TRN pathway therefore serves as an emotional gatekeeper that can render the cortex ‘blind’ to neutral signals.

The robust amygdala-TRN pathway likely serves as a powerful conduit for affectively-charged information to restrict attention and bias decision-making [1,3]. Highly salient affective signals can change how one integrates available information, pushing the decision-making process towards a ‘black-and-white’ mode that is less sensitive to nuanced signals, such as the actual probabilities of success and failure. Probabilistic decision-making is strongly biased by the salience of the decision's outcome, whether positive or negative. ‘Affect-rich’ choices, accompanied by enhanced activation in the amygdala and thalamus, reflect the actual probabilities of success and failure less than ‘affect-poor’ choices [3]. In the framework of the EmGate model, such phenomena may be described as dual facets of top-down bias: (1) amplification of attention to outcome information, and (2) simultaneous suppression of attention to other pertinent information, such as the probability of the outcome. The result is often warped decision-making. Such a mechanism may contribute to the well-known tendency of gamblers to discount key information, including the probabilities of gain and loss: the affective salience of a possible big win may blind gamblers to the fact that the chance of winning is very small.

The amygdala-TRN pathway may also serve as a powerful mediator of the ‘framing effect’ on decision-making, which depends on loss aversion, which is strongly linked with activity in the amygdala [1]. The EmGate model demonstrates how the amygdala-TRN pathway can select signals that are made available to cortical areas for cognitive processing, and suppress all other signals. The robustness of the amygdalar pathways suggests that they are particularly well-suited to convey signals related to survival [5,28,29]. The amygdala-TRN pathway may contribute to shifting the ‘operating point’ of decision-making along a spectrum from debilitating caution to reckless overconfidence [59].

But rather than having a purely ‘irrational’ influence on decision-making [1,60], emotional modulation of attention may also serve to set clear priorities and therefore ‘non-linearize’ the system in adaptive, context-sensitive ways. Decisions based on deliberative evaluation of all available evidence may not always be sufficiently “fast and frugal”, and so it may often be useful for the emotional system to filter out all but the most important information [61]. Our simulations show that in the bottom-up mode, the amygdala ‘raises the alarm’ when salient information is present in the environment, contributing evidence in support of a particular behavioral plan. The importance of such emotional ‘drive’ is supported by the observation that lesions of the amygdala and pOFC lead to severe dysfunction of decision-making [62]. Our simulations provide support for the idea that the amygdala-TRN projection can function as a relevance detector, highlighting only the stimuli that are consistent with a pre-selected plan and suppressing everything else [57].

In addition to the distinctive sensory selection process made possible by the amygdala-TRN pathway, our simulations reveal the importance of local inhibition in planning-related cortices in resetting the decision-making process and thereby facilitating shifts in attention in response to changes in bottom-up contingency. Inhibition of the plan cortical neurons by local interneurons interrupts excitatory cortico-thalamic feedback, preventing persistence of a chosen plan. We hypothesize that two sources of excitation are necessary for appropriate resetting by these interneurons: expectation confirmation and expectation violation. When an affective expectation is met, expectation confirmation allows the system to move on to a new behavioral plan.When an affective expectation fails to materialize, expectation violation allows the system to move on instead of becoming ‘stuck’ or ‘frustrated’.

Our simulations also suggest an important role for amygdalar inhibitory interneurons and their excitatory inputs in attentional flexibility. Signals that represent stimulus-specific confirmation of expectation or violation of expectation serve as inputs to these interneurons, which inhibit nearby projection neurons in the amygdala and thus prevent fixation of emotional attention. The importance of amygdalar interneurons in shifting emotional attention is a testable prediction of our model. A weakening of the inhibition provided by amygdalar interneurons can lead to excessive attentional fixation. Inhibition within the amygdala can support flexible shifting of attention among all the stimuli that are relevant to the ongoing plan. Without it, attention becomes fixated on the first available relevant stimulus and cannot shift until the plan shifts.

### The relationship between the EmGate model and related networks

The simulations reveal that inhibitory interneurons in cortex and amygdala facilitate flexible decision-making and attentional shifting. Their excitatory inputs are therefore pivotal to these forms of flexibility. We have employed two types of excitatory input to the interneurons: expectation confirmation and expectation violation. We assume that these signals can be computed locally (Fig 1 Insets E and F), because nearby principal neurons provide information sufficient for this purpose. Plan cortical activity can serve as a proxy for plan-related expectation. Similarly, activity in the lateral amygdala can serve as a proxy for stimulus-related expectation. But there is no reason to assume that expectation-related processing only occurs in these regions. The expectation confirmation and violation signals implemented here represent processes that likely involve multiple brain regions. It is possible that the excitatory inputs to the amygdalar interneurons are supplied at least partly by PFC subregions which engage in two-way communication with BA. A cortical origin for these inputs is supported by evidence that OFC and ACC show activity related to expectations and their violation [63,64]. Both OFC and ACC have bidirectional connections with BLA [16,17].

Neuromodulatory dopamine signals such as those emerging from the ventral tegmental area are also pertinent both as appetitive reinforcement signals and as signals that redirect attention [65]. Although these signals are stimulus- and learning-dependent, they do not convey specific stimulus properties. Rather, dopamine bursts signal unexpected rewards and unexpected cued opportunities for reward–both powerful redirectors of attention. In primate prefrontal cortex, dopamine facilitates inhibition mediated by GABAergic interneurons [66], which our model implicates in flexible shifts of attention, and in rodent prefrontal cortex, a deficiency of dopaminergic regulation of such interneurons results in a loss of cognitive flexibility [67].

The effects on attention mediated by the amygdala-TRN pathway may act in parallel with other pathways, such as the projection from the central nucleus of the amygdala to the basal forebrain [68]. The entire cortical mantle receives cholinergic projections from neurons in the basal forebrain [69–73]. Acetylcholine plays an important modulatory role in attention and other cognitive processes [74–79]. Projections from the amygdala to the basal forebrain are therefore in a position to modulate cholinergic processing across the cortex, thereby affecting attentional and preattentional mechanisms. In a recent study in rhesus macaques, simultaneous recording in the amygdala and the basal forebrain showed that neurons in the basal forebrain respond similarly to amygdalar neurons, firing selectively for reward-predictive stimuli [80]. Neurons in the amygdala tracked trial-to-trial fluctuations in spatial attention, whereas basal forebrain neurons did not. The close relationship between phasic amygdalar activity and allocation of attention is consistent with our simulation results. The amygdalar projections to basal forebrain may contribute to a more broad, tonic form of affective expectation that modulates the cholinergic effects of the basal forebrain on other brain areas. Changes in acetylcholine levels can result in changing sensory tuning curves and other receptive field properties in cortical and subcortical areas [75]. These changes may act in cooperation with the faster attentional effects mediated by the amygdala-TRN projection. The amygdala-basal forebrain projection can also result in feedback onto the amygdala. Cholinergic input from the basal forebrain can elevate the firing of amygdalar neurons to emotionally salient stimuli and suppress firing of neutral stimuli [81]. Such effects are likely to act in synergy with the mechanisms simulated here.

### Symptoms of psychiatric disorders viewed as dysfunctions in the circuit for emotional attention

The EmGate model allows us to propose three types of mechanisms that can mediate the attentional symptoms of distinct psychiatric disorders. The first mechanism involves top-down excitatory drive to a particular behavioral plan. Disorders of top-down drive may result in abnormally strong or weak top-down drive to the amygdala, leading to abnormally strong or weak attentional fixation on particular classes of stimuli. Patients with phobias, for example, exhibit excessive top-down bias towards fear-related stimuli [82], which co-occurs with hyperactivation of the amygdala [83]. Autism appears to involve a weakened drive towards emotionally salient stimuli such as expressive faces [84], also suggested by weak long-range connections [85–87]. The second mechanism involves dysregulation of inhibition in planning-related prefrontal areas. Our simulations suggest that weak inhibition can result in inability to shift behavioral plans in response to changing contingencies, accompanied by restriction of attention to the salient stimuli that are consistent with the uninhibited plan. Fast inhibition can instead result in rapid shifting of behavioral plans and sensory attention. This may contribute to the impulsivity and distractibility observed in attention deficit hyperactivity disorder (ADHD) [88]. The third mechanism involves dysregulation of the inhibitory interneurons in the amygdala. Dysregulation may be caused by damage to the interneurons themselves, or by alterations to the pathways conveying excitatory expectation-related signals from cortex to the inhibitory interneurons. Slow or weak inhibition from amygdalar interneurons may result in excessive focus on salient stimuli even long after these stimuli have been removed from the environment. In other words, the EmGate model suggests that dysfunctional emotional attention can result from abnormal top-down drive, or from loss of flexibility. The two mechanisms are not mutually exclusive, and may co-occur in some disorders. Psychiatric disorders likely involve dysfunction in a variety of brain areas, so the mechanisms suggested by the model may act in parallel with abnormalities elsewhere.

### Conclusion

Signals pertaining to emotional salience provide crucial information for flexible allocation of attention. The recently discovered pathway from the amygdala to TRN is strategically positioned to link affective salience signals with a key attentional system [5]. We developed a novel computational neural network model, the Emotional-Gatekeeper (EmGate) that embeds this pathway in a wider network that subserves emotional attention. The model demonstrates two kinds of attentional gatekeeping based on emotions: bottom-up attention that tracks the salience of current stimuli, and top-down attention that focuses on stimuli that are relevant to a chosen plan and suppresses irrelevant stimuli. The model highlights the unique ability of the amygdala-TRN pathway to filter the information available for subsequent cortical decision-making and other cognitive processes. This mechanism is likely to be one of the key conduits for phenomena such as biased decision-making, affective framing, emotion-induced blindness, and relevance detection. The model also suggests three mechanisms that may underlie the symptoms of emotional disorders that pertain to decision-making and attention: dysfunctional top-down modulation of the output neurons of the amygdala, dysregulation of inhibitory neurons in planning-related prefrontal cortex, and dysregulation of inhibitory neurons in the basal amygdala. The model’s prediction of a key role for interneurons in the cortex and in the amygdala in flexible attention and decision-making can be tested in animal models using techniques such as lesions, optogenetic stimulation and pharmacological manipulation.

## Methods

### Mathematical specification of the rate model

We implement the model as a rate-coded system, which allows us to focus on the properties of the network that depend more on connectivity than fine-grained biophysical details. We assume that each model neuron’s activity *x* obeys the same type of differential equation:

$$\tau \frac{dx}{dt}=-Ax+\left(B-x\right)E-\left(x+C\right)I$$ (1)

where *E* denotes the excitatory input, *I* denotes the inhibitory input, *A* is a passive decay rate, *B* is the maximum activity (*B*>0), and −*C* is the minimum activity (*C*≥0). The time constant of integration is τ. The excitatory and inhibitory inputs for each type of model neuron are specified in Table 1. Square brackets with a superscript + indicate positive rectification (i.e., if *a*≥0, [*a*]^+^ = *a*, else [*a*]^+^ = 0). Symbols *x* and *y* denote neural activity values, and symbols *W* denote connection weights. The symbol *H(*.*)* denotes the Heaviside step function, which takes the value of 1 if its argument is strictly greater than zero, and zero otherwise. The weights that are not subject to synaptic modification are specified in Table 2. The simulations are robust with respect to perturbations of the constant connection weights. Model variants in which these weights are individually perturbed in the ranges specified in Table 2 display qualitatively similar properties. Outside these ranges the qualitative behavior breaks down. Parameters were chosen so that the suppressive effects of the BA-TRN projection were clearly visible in the simulations. Other parameter choices can diminish the all-or-nothing nature of attentional suppression. For example, smaller values of the weights linking TRN with thalamus ($W_{ij}^{SR\to ST}$ and $W_{pq}^{PR\to PT}$) lead to weakened off-surround inhibition of competing CRT loops.

**Table 1:** Model neural activities.

Term	Symbol (x)	Excitatory input (E)	Inhibitory input (I)
Sensory thalamus	$x_{i}^{ST}$	$S_{i}+W^{SC\to ST}x_{i}^{SC}+W^{BA\to ST}({\left[x_{i}^{BA_{1}}\right]}^{+}+{\left[x_{i}^{BA_{2}}\right]}^{+})$	$\sum_{j=1}^{N}W_{ji}^{SR\to ST}{\left[x_{j}^{SR}\right]}^{+}$
Sensory cortex	$x_{i}^{SC}$	$W^{ST\to SC}{\left[x_{i}^{ST}\right]}^{+}+W^{BA\to SC}({\left[x_{i}^{BA_{1}}\right]}^{+}+{\left[x_{i}^{BA_{2}}\right]}^{+})$	0
Sensory TRN	$x_{i}^{SR}$	$W^{SC\to SR}x_{i}^{SC}+W^{ST\to SR}{\left[x_{i}^{ST}\right]}^{+}+W^{BA\to SR}({\left[x_{i}^{BA_{1}}\right]}^{+}+{\left[x_{i}^{BA_{2}}\right]}^{+})$	$\sum_{j=1}^{N}W_{ji}^{SR\to SR}{\left[x_{j}^{SR}\right]}^{+}$
LA	$x_{i}^{LA_{q}}$	$W_{i}^{ST\to LA_{q}}{\left[x_{i}^{ST}\right]}^{+}$	0
BA	$x_{i}^{BA_{q}}$	$W^{LA\to BA_{q}}{\left[x_{i}^{LA_{q}}\right]}^{+}+W_{qi}^{PC\to BA_{q}}{\left[x_{q}^{PC}-\Gamma^{PCB}\right]}^{+}$	$W^{yB}y_{i}^{BAIN_{q}}$
BA interneuron	$y_{i}^{BAIN_{q}}$	$W^{Lcy}{\left[x_{qi}^{Lc}\right]}^{+}+W^{Lvy}{\left[x_{qi}^{Lv}\right]}^{+}$	0
Amygdala expectation confirmation	$x_{qi}^{Lc}$	$W^{Lc}{\left[x_{i}^{LA_{q}}\right]}^{+}R_{q}$	0
Amygdala expectation violation	$x_{qi}^{Lv}$	$W^{Lv}{\left[x_{i}^{LA_{q}}-\Gamma^{L}\right]}^{+}+W^{vv}{\left[x_{qi}^{Lv}-\Gamma^{v}\right]}^{+}$	$W^{Lcv}x_{qi}^{Lc}+W^{Lyv}{\left[y_{qi}^{Lvr}-\Gamma^{yv}\right]}^{+}$
Amygdala expectation violation reset	$y_{qi}^{Lvr}$	$W^{Ly}{\left[x_{qi}^{Lv}\right]}^{+}+W^{yy}{\left[y_{qi}^{Lv}-\Gamma^{y}\right]}^{+}H\left(x_{qi}^{Lv}-\Gamma^{r}\right)$	0
Plan thalamus	$x_{q}^{PT}$	$M_{q}+W^{PC\to PT}{\left[x_{q}^{PC}-\Gamma^{PCT}\right]}^{+}+W^{BA\to PT}\sum_{j=1}^{N}{\left[x_{j}^{BA_{q}}\right]}^{+}$	$\sum_{p=1}^{2}W_{pq}^{PR\to PT}{\left[x_{p}^{PR}\right]}^{+}$
Plan cortex	$x_{q}^{PC}$	$W^{PT\to PC}{\left[x_{q}^{PT}\right]}^{+}+W^{BA\to PC}\sum_{j=1}^{N}{\left[x_{j}^{BA_{q}}\right]}^{+}$	$W^{yP}y_{q}^{PCIN}$
Plan TRN	$x_{q}^{PR}$	$W^{PC\to PR}x_{q}^{PC}+W^{PT\to PR}{\left[x_{q}^{PT}\right]}^{+}+W^{BA\to PR}\sum_{j=1}^{N}{\left[x_{j}^{BA_{q}}\right]}^{+}$	$\sum_{p=1}^{2}W_{pq}^{PR\to PR}{\left[x_{p}^{PR}\right]}^{+}$
Plan cortex interneuron	$y_{q}^{PCIN}$	$W^{Pcy}{\left[x_{q}^{Pc}\right]}^{+}+W^{Pvy}{\left[x_{q}^{Pv}\right]}^{+}$	0
Plan cortex expectation confirmation	$x_{q}^{Pc}$	$W^{Pc}{\left[x_{q}^{PC}\right]}^{+}R_{q}$	0
Plan cortex expectation violation	$x_{q}^{Pv}$	$W^{Pv}{\left[x_{q}^{PC_{}}-\Gamma^{P}\right]}^{+}+W^{vv}{\left[x_{q}^{Pv}-\Gamma^{v}\right]}^{+}$	$W^{Pcv}x_{q}^{Pc}+W^{Pyv}{\left[y_{q}^{Pvr}-\Gamma^{yv}\right]}^{+}$
Plan cortex expectation violation reset	$y_{q}^{Pvr}$	$W^{Py}{\left[x_{q}^{Pv}\right]}^{+}+W^{yy}{\left[y_{q}^{Pv}-\Gamma^{y}\right]}^{+}H\left(x_{q}^{Pv}-\Gamma^{v}\right)$	0

**Table 2:** Parameter values for the rate-coded model.

Connection weight	Value	Range	Constant	Value
*W* ^*SC*→*ST*^	0.8	0.01–1.00	Γ^*PCB*^	1
*W* ^*ST*→*SC*^	0.8	0.01–1.00	Γ^*PCT*^	0.10
*W* ^*SC*→*SR*^	0.15	0.01–0.30	Γ^*L*^	0.03
*W* ^*ST*→*SR*^	0.1	0.01–0.40	Γ^*v*^	0.10
*W*_*ij*_^*SR*→*ST*^ (*i* ≠ *j*)	3	1.50–4.50	Γ^*yv*^	0.10
*W*_*ij*_^*SR*→*SR*^ (*i* ≠ *j*)	3	1.50–9.00	Γ^*y*^	0.10
*W* ^*BA*→*ST*^	0.01	0.001–0.030	Γ^*r*^	1
*W* ^*BA*→*SC*^	0.01	0.001–0.030	Γ^*P*^	2
*W* ^*BA*→*SR*^	0.25	0.125–0.375	Γ^*ST*^	0.75
*W* ^*PC*→*PT*^	9	6–16	Γ^*BA*^	0.10
*W* ^*PT*→*PC*^	9	6–16	*B* ^*ST*→*LA*^	1
*W* ^*PC*→*PR*^	0.5	0.05–2.00	*B* ^*PC*→*BA*^	1
*W* ^*PT*→*PR*^	0.1	0.01–0.40	*τ* ^*ST*,*SC*,*SR*,*LA*,*BA*,*PT*,*PC*,*PR*,*BAIN*,*PCIN*,*w*^	0.05
*W*_*pq*_^*PR*→*PT*^ (*p* ≠ *q*)	1	0.10–4.00	*τ* ^*Lc*,*Pc*^	0.25
*W*_*pq*_^*PR*→*PR*^ (*p* ≠ *q*)	1	0.10–4.00	*τ* ^*Lv*,*Lvr*^	3.33
*W* ^*BA*→*PT*^	0.5	0.05–1.50	*τ* ^*Pv*,*Pvr*^ (slow reset)	12.50
*W* ^*BA*→*PC*^	0.5	0.05–1.50	*τ* ^*Pv*,*Pvr*^ (fast reset)	2.50
*W* ^*BA*→*PR*^	1	0.01–4.00	*A* ^*ST*^	1
$W^{LA\to BA_{q}}$	3	2.25–3.75	*A* ^*SC*,*PC*,*LA*,*y*^	100
*W* ^*yB*^	30	22.50–45	*A* ^*SR*,*PR*,*BA*^	10
*W* ^*Lcy*^	3	0.03–12	*A* ^*PT*^	5
*W* ^*Lvy*^	1	0.75–1.50	*A* ^*r*^	200
*W* ^*Lc*^	5	0.05–10.00	*A* ^*Lv*,*Pv*^	0.20
*W* ^*Lv*^	1	0.50–1.10	*A* ^*Lc*,*Pc*^	3
*W* ^*Ly*^	5	1.25–12.50	*B*	10
*W* ^*w*^	20	15–20	*C*	10
*W* ^*yy*^	80	40–80	*S*_*i*_, *R*_*q*_ (when present)	1
*W* ^*Lcv*^	10	1–40	*M*_*q*_ (when present)	160
*W* ^*Lyv*^	10	5–40		
*W* ^*yP*^	60	45–120		
*W* ^*Pcy*^	5	1.25–15.00		
*W* ^*Pvy*^	2	1.50–5.00		
*W* ^*Pc*^	10	1–30		
*W* ^*Pv*^	0.1	0.075–0.40		
*W* ^*Py*^	1	0.25–1.25		
*W* ^*Pcv*^	10	1–40		
*W* ^*Pyv*^	8	6–20		

The thalamic, cortical and TRN components of the sensory map each contain *N* (= 10) model neurons. *S*_*i*_ denotes the *i*th sensory input (out of a total of *N* inputs) arriving at the sensory map. $W_{ji}^{SR\to ST}$ is the connection strength between the *j*th TRN neuron and the *i*th thalamic neuron. $W_{ji}^{SR\to SR}$ is the connection strength between the *j*th TRN neuron and the *i*th TRN neuron. These weights are zero for all subscripts *i* = *j*, which enables open-loop connectivity. Similarly, $W_{pq}^{PR\to PT}$ and $W_{pq}^{PR\to PR}$ are zero when *p* = *q*. When distractors are presented to a sensory thalamic neuron the corresponding *S*_*i*_ is a stochastic input.

In both LA and BA one subgroup of *N* neurons represents appetitive stimuli, and another set of *N* neurons represents aversive stimuli. Thus subgroups *LA*_1_ and *BA*_1_ correspond to appetitive stimuli, and subgroups *LA*_2_ and *BA*_2_ correspond to aversive stimuli. LA serves as the input station of the amygdala, and BA serves as the output station, projecting back to the sensory TRN and also to the thalamic neurons of the plan map. Sensory information from the thalamus ($x_{i}^{ST}$) is mapped topographically to LA. $W_{i}^{ST\to LA_{q}}$ is the connection strength between the *i*th sensory thalamic neuron and the *i*th *LA*_*q*_ neuron. Index *q* is 1 for appetitive neurons and 2 for aversive neurons. Cells in LA project topographically to BA. $W_{qi}^{PC\to BA_{q}}$ is the connection strength between the *q*th plan cortical neuron and the *i*th *BA*_*q*_ neuron. $W^{LA\to BA_{q}}$ is the constant connection strength between each *LA*_*q*_ neuron and the corresponding *BA*_*q*_ neuron.

In addition to the principal neurons, there is one inhibitory interneuron for each BA principal neuron. The term $y_{i}^{BAIN_{q}}$ denotes the activity of an inhibitory interneuron that mediates a reset process for the *i*th *BA*_*q*_ neuron, and is excited by an expectation confirmation signal $x_{qi}^{Lc}$, and an expectation violation signal $x_{qi}^{Lv}$. The activity of each LA neuron serves as a form of expectation that the corresponding stimulus will result in the associated reinforcement. The expectation confirmation signal $x_{qi}^{Lc}$ is a measure of whether LA activity $x_{i}^{LA_{q}}$ co-occurs with the corresponding reinforcement signal *R*_*q*_. The expectation violation signal $x_{qi}^{Lv}$ builds up due to the prolonged failure of LA activity to co-occur with the reinforcement signal *R*_*q*_. This build-up is governed by self-excitation, which is weighted by *W* ^*yy*^. It is inhibited by the excitation confirmation signal, and is also rapidly reset by an inhibitory activity $y_{qi}^{Lvr}$. The inhibitory activity $y_{qi}^{Lvr}$ is triggered by $x_{qi}^{Lv}$. Its level builds up due to self-excitation weighted by *W* ^*vv*^. Self-excitation shuts off if the input $x_{qi}^{Lv}$ falls below a threshold Γ^*Lvr*^. A hard nonlinearity is introduced for the expectation violation signal, ensuring that it is non-negative following resetting by the inhibitory activity. The time constants *τ* ^*Lv*,*Lvr*^ determine the rates of build-up and recovery of the expectation violation signal.

The pair of equations governing the expectation violation signal are designed to model in a phenomenological manner a gradual growth of excitation that quickly shuts off after reaching a peak value. The equations are parametrized to allow simple manipulation of the timing of onset of the expectation violation signal. A more detailed model of expectation could replace these phenomenological equations, incorporating additional factors such as learning of the expected time of reinforcement delivery.

The 2 sets of *N* neurons in BA project to the plan map. The plan map consists of 2 possible plans, and for each plan there is a corresponding thalamic, TRN and cortical neuron. The term *M*_*q*_ is an extrinsic motivational drive that can bias the competition between behavioral plans. As in the amygdala, an inhibitory interneuron with activity $y_{q}^{PCIN}$ triggers resetting of each plan cortical activity. These interneurons receive expectation confirmation and violation signals that have the same dynamic properties as those in the amygdala. The activity of each plan cortical neuron represents the expectation that the corresponding plan will be accompanied by the delivery of the associated reinforcement. The main difference between the amygdalar expectation and the plan cortical expectation is that the amygdalar variant is stimulus-specific, whereas the plan cortical variant is plan-specific. The time constants *τ* ^*Pv*,*Pvr*^ determine the rates of build-up and recovery of the expectation violation signal. The speed of resetting in Fig 3 and Fig 4 is determined by the values of these two time constants. Slow resetting (Fig 3) is simulated using larger values, and fast resetting is simulated using smaller values (Fig 4).

Two sets of synaptic weights are subject to reinforcement-driven LTP: $W_{i}^{ST\to LA_{q}}$, and $W_{qi}^{PC\to BA_{q}}$. We use two reinforcement signals: *R*_1_ which signals the occurrence of an appetitive event such as food delivery, and *R*_2_ which signals the occurrence of an aversive event such as an airpuff. Reinforcement signals are square pulses, i.e., *R*_*q*_ = 1 when a reinforcement is delivered, and *R*_*q*_ = 0 otherwise.

The connection weights linking sensory thalamus to *LA*_*q*_ ($W_{i}^{ST\to LA_{q}}$) are determined by:

$$\tau^{w}\frac{d}{dt}W_{i}^{ST\to LA_{q}}=\left(B^{ST\to LA}-W_{i}^{ST\to LA_{q}}\right){\left[x_{i}^{ST}-\Gamma^{ST}\right]}^{+}R_{q}$$ (2)

where the learning is pre-synaptically gated by the activity of the corresponding sensory thalamic neuron activity $x_{i}^{ST}$.

The connection weights linking plan cortex to BA ($W_{qi}^{PC\to BA_{q}}$) are determined by:

$$\tau^{w}\frac{d}{dt}W_{qi}^{PC\to BA_{q}}=\left(B^{PC\to BA}-W_{qi}^{PC\to BA_{q}}\right){\left[x_{i}^{BA_{q}}-\Gamma^{BA}\right]}^{+}{\left[x_{q}^{PC}\right]}^{+}R_{q}$$ (3)

where the learning is pre-synaptically gated by the activity of the *q*th plan cortical neuron activity $x_{q}^{PC}$ and also post-synaptically gated by the activity of the *i*th BA principal neuron activity $x_{i}^{BA_{q}}$. Index *q* takes the value 1 for the appetitive plan, and 2 for the aversive plan. The terms *B* ^*ST*→*LA*^ and *B* ^*PC*→*BA*^ are constants that determine the maximum possible weight of $W_{i}^{ST\to LA_{q}}$ and $W_{qi}^{PC\to BA_{q}}$, respectively. In a more biophysically detailed version of the model, the presynaptic activities, which here overlap the reinforcement signals in time, could be replaced by eligibility traces of prior presynaptic activities, which could then be allowed to reset before reinforcement delivery. For a precedent in a rate model, see section 7 of [89]; for a spiking model precedent, see [90].

### Mathematical specification of the spiking model

We also simulated a spiking version of the EmGate model, in which the rate-coded model neurons are replaced by spiking Izhikevich neurons [58]. The simulations reveal that the qualitative behavior of the model is robust with respect to choice of model neuron. Each Izhikevich neuron is governed by the following coupled differential equations:

$$\tau_{s}\frac{dv}{dt}=0.04v^{2}+5v+140-u+E-I$$ (4)

$$\tau_{s}\frac{du}{dt}=a(bv-u)$$ (5)

along with an auxiliary after-spike resetting:

$$\text{if}\;v\geq 30,\;\text{then}\;\{\begin{array}{c} v\overset{}{\leftarrow }c\\ u\overset{}{\leftarrow }u+d \end{array}$$ (6)

where *v* represents the voltage of the neuron, *u* is a recovery variable, and *a*, *b*, *c*, and *d* are parameters that facilitate approximating various types of spiking neuron. A detailed discussion of the role of each parameter is found in [58]. *E* and *I* are the excitatory and inhibitory inputs, respectively. The integration time constant is *τ*_*s*_.

Connectivity in the model is shown in Fig 1. The inputs *E* and *I* for each neuron type are the same as in Table 1. However, the rate-model’s rates of activation *x* and *y* in Table 1 and Eqs (1), (2) and (3) cannot be directly replaced by the corresponding voltages *v* of the spiking neurons. A model of synaptic activity must be used to translate the presynaptic spikes of voltages *v* into postsynaptic potentials. To model post-synaptic potentials we use the saturating differentials (SD) spike-dependent signal *g*_*SD*_, which is governed by the following coupled differential equations [91].

$$\tau_{s}\frac{dT}{dt}=(1-T)K-\frac{T}{\tau_{rise}}$$ (7)

$$\tau_{s}\frac{dg_{SD}}{dt}=\left(\frac{\tau_{fall}+\tau_{rise}}{\tau_{fall}}\right)\left[\frac{2}{\tau_{rise}}\left(1-g_{SD}\right)T-\frac{g_{SD}}{\tau_{fall}}\right]$$ (8)

The intermediate variable *T* is triggered by the arrival of a discrete spike *K*, and can be interpreted as the presence of transmitter in the synaptic cleft. The spike variable *K* takes the value of 1 at the moment the voltage *v* goes above a threshold (20), and is zero at all other times. The parameters *τ*_*rise*_ and *τ*_*fall*_ determine the rise and fall times, respectively, of the post-synaptic potential. The parameter *τ*_*s*_ governs the rate of integration in Eqs (4–8). To complete the spiking model we replace the terms *x* and *y* with the corresponding value of the postsynaptic potential *g*_*SD*_. These net postsynaptic potentials *g*_*SD*_ can be interpreted as accomodating several temporal factors, such as conduction delays and the rapid and medium-term components of postsynaptic potentials.

For the principal neurons, the values of *a*, *b c* and *d* are chosen to simulate the regular spiking neuron type (0.02, 0.2, -65, and 8, respectively). All inhibitory neurons have values of *a*, *b*, *c*, and *d* chosen to simulate the fast spiking neuron type (0.1, 0.2, -65, and 2, respectively). The rise time *τ*_*rise*_ is 4 milliseconds, and the fall time *τ*_*fall*_ is 40 milliseconds.

The expectation confirmation and violation signals are not modeled as spiking neurons. Instead they are modeled at a more phenomenological level, using the same class of equations as in the rate model. This allows for more direct control over the time course of inhibition triggered by expectation confirmation and violation. The parameters of the Izhikevich model and the SD signal do not allow for simple modeling of signals that build up slowly and reset themselves rapidly. A more elaborated spiking model would need to incorporate additional mechanisms beyond those captured in Eqs (4) to (8). Synaptic connection weights in the spiking model are distinct from those in the rate-coded model, due to the differences in the properties of the model neurons used. These values are shown in Table 3.

**Table 3:** Parameter values for the spiking model.

Connection weight	Value	Range	Constant	Value
*W* ^*SC*→*ST*^	40	4–160	Γ^*PCB*^	0
*W* ^*ST*→*SC*^	40	4–160	Γ^*PCT*^	0
*W* ^*SC*→*SR*^	1	0.01–4.00	Γ^*L*^	0.1
*W* ^*ST*→*SR*^	1	0.01–4.00	Γ^*v*^	0.1
*W*_*ij*_^*SR*→*ST*^ (*i* ≠ *j*)	100	75–200	Γ^*yv*^	0.1
*W*_*ij*_^*SR*→*SR*^ (*i* ≠ *j*)	80	60–240	Γ^*y*^	0.1
*W* ^*BA*→*ST*^	0.3	0.03–1.20	Γ^*r*^	1.0
*W* ^*BA*→*SC*^	0.3	0.03–1.20	Γ^*P*^	0.5
*W* ^*BA*→*SR*^	80	40–120	Γ^*ST*^	0
*W* ^*PC*→*PT*^	200	100–300	Γ^*BA*^	0
*W* ^*PT*→*PC*^	200	150–220	*B* ^*ST*→*LA*^	100
*W* ^*PC*→*PR*^	100	25–300	*B* ^*PC*→*BA*^	40
*W* ^*PT*→*PR*^	10	1–40	*τ* ^*w*^	0.025
*W*_*pq*_^*PR*→*PT*^ (*p* ≠ *q*)	16	4–20	*τ* ^*s*^	0.001
*W*_*pq*_^*PR*→*PR*^ (*p* ≠ *q*)	16	4–64	*τ* ^*Lc*,*Pc*^	0.25
*W* ^*BA*→*PT*^	20	15–25	*τ* ^*Lv*,*Lvr*^	3.33
*W* ^*BA*→*PC*^	5	0.05–20.00	*τ* ^*Pv*,*Pvr*^ (slow reset)	10
*W* ^*BA*→*PR*^	12	1.2–48.0	*τ* ^*Pv*,*Pvr*^ (fast reset)	1.33
$W^{LA\to BA_{q}}$	100	25–400	*S*_*i*_, *R*_*q*_ (when present)	1
*W* ^*yB*^	70	60–105	*M*_*q*_ (when present)	160
*W* ^*Lcy*^	50	5–200		
*W* ^*Lvy*^	3	2.25–9.00		
*W* ^*Lc*^	1	0.1–3.0		
*W* ^*Lv*^	1	0.9–1.5		
*W* ^*Ly*^	1	0.50–1.25		
*W* ^*w*^	5	4.5–7.5		
*W* ^*yy*^	100	50–200		
*W* ^*Lcv*^	25	2.5–75		
*W* ^*Lyv*^	10	5–12.5		
*W* ^*yP*^	180	170–360		
*W* ^*Pcy*^	100	25–300		
*W* ^*Pvy*^	10	2.5–20.0		
*W* ^*Pc*^	0.1	0.025–0.300		
*W* ^*Pv*^	1	0.75–2.00		
*W* ^*Py*^	1	0.25–1.50		
*W* ^*Pcv*^	25	2.5–100.00		
*W* ^*Pyv*^	10	5–15		

### Simulation details

#### Pavlovian conditioning simulations

Each simulation is run for *T* = 160000 time steps, which is the equivalent of 16 seconds of real time. This period is divided into four epochs of equal duration. The first two epochs of each simulation run are conditioning phases. Each stimulus *S*_*i*_ presented is a rectangular pulse of activation with amplitude 1 and duration 1000 time steps. In the first epoch, CS1 and CS2 are each presented 4 times, in alternation. After each presentation of CS1 or CS2, the reinforcer *R*_1_ is presented after a delay (Δ*t* = 250 time steps). In the second epoch, CS2 is presented alone 8 times. The reinforcer *R*_2_ follows CS2 after the same delay (Δ*t*). The third and fourth epochs of each simulation run are testing phases. During these phases, CS1, CS2 and CS3 are presented in an alternate pattern. No reinforcement is delivered during the testing phases. Each reinforcement signal *R*_1_ and *R*_2_ is a rectangular pulse of activity with amplitude 1 and duration 750 time steps. Distractor stimuli are delivered in the third and fourth epochs, and each has amplitude 1 and duration 600 time steps. All activity values *x* and modifiable connection weights *W* are initialized to 0 at the start of each simulation run. In the spiking simulations, voltages are initialized to -65 millivolts. We do not use extrinsic resetting within a simulation run, so the system switches its attention and its behavioral plan using the inhibitory mechanisms available. Extrinsic motivational drives *M*_1_ and *M*_2_ vary depending on the simulation run. Each takes a value of either 0 (no motivation) or 160 (high motivation) during Testing Phases 2. Parameters are specified in Tables 2 and 3. All simulations were performed in MATLAB (R2013b). The equations were integrated using the forward Euler method, with a step size *h* = 0.0001 seconds. Image annotations were added using Inkscape.

#### “Emotion-induced blindness” simulations

We also simulated a version of the “attentional rubbernecking” paradigm [4]. The task is based on rapid serial visual presentation (RSVP), in which each trial consists of a non-overlapping sequence of stimuli, each presented for a duration of 100 milliseconds. One of these stimuli is a target stimulus that must be correctly identified. Emotionally salient stimuli are also included in the sequence. When an emotional stimulus precedes the neutral target stimulus, it can impair detection if the lag between offset of one stimulus and onset of the next is small. In our simplified version of this paradigm, each trial consist of a stimulus (S1) followed by the neutral target stimulus (S2) that must be detected. Detection occurs successfully if the sensory cortical activity corresponding to S2 crosses a threshold. The threshold varies from trial-to-trial, and is drawn from a uniform distribution bounded between 0.05 and 0.35. This variability is meant to capture all sources of stochasticity that have not been explicitly modeled here, such as fluctuating levels of arousal. The stimuli are separated by a time lag. Two time lags are used, a short lag (500 time steps, or 50 milliseconds) and a long lag (4000 time steps, or 400 milliseconds). There are 20 testing trials for each time lag. The first five trials are shown in Fig 11. Prior conditioning lasts for 200000 time steps. The total time for 20 testing trials is 200000 time steps (20 seconds). Other parameters are as in the simulations for Figs 3–6.
